# Innate TCRβ-chain engagement drives human T cells toward distinct memory-like effector phenotypes with immunotherapeutic potentials

**DOI:** 10.1126/sciadv.adj6174

**Published:** 2023-12-06

**Authors:** Pierre Vantourout, Josephine Eum, María Conde Poole, Thomas S. Hayday, Adam G. Laing, Khiyam Hussain, Rosamond Nuamah, Shichina Kannambath, Jacques Moisan, Allart Stoop, Sebastiano Battaglia, Roya Servattalab, Jonathan Hsu, Andrew Bayliffe, Madan Katragadda, Adrian C. Hayday

**Affiliations:** ^1^ Peter Gorer Department of Immunobiology, School of Immunology and Microbial Sciences, King’s College London, London, SE1 9RT, UK.; ^2^ Immunosurveillance Laboratory, The Francis Crick Institute, London, NW1 1AT, UK.; ^3^ NIHR BRC Genomics Research Platform, Guy's and St Thomas' NHS Foundation Trust, King's College London School of Medicine, Guy's Hospital, London, SE1 9RT, UK.; ^4^ Marengo Therapeutics, Cambridge, MA 02139, USA.; ^5^ Bridge Informatics, Salem, MA, 01970, USA.

## Abstract

Clonotypic αβ T cell responses to cargoes presented by major histocompatibility complex (MHC), MR1, or CD1 proteins underpin adaptive immunity. Those responses are mostly mediated by complementarity-determining region 3 motifs created by quasi-random T cell receptor (TCR) gene rearrangements, with diversity being highest for TCRγδ. Nonetheless, TCRγδ also displays nonclonotypic innate responsiveness following engagement of germline-encoded Vγ-specific residues by butyrophilin (BTN) or BTN-like (BTNL) proteins that uniquely mediate γδ T cell subset selection. We now report that nonclonotypic TCR engagement likewise induces distinct phenotypes in TCRαβ^+^ cells. Specifically, antibodies to germline-encoded human TCRVβ motifs consistently activated naïve or memory T cells toward core states distinct from those induced by anti-CD3 or superantigens and from others commonly reported. Those states combined selective proliferation and effector function with activation-induced inhibitory receptors and memory differentiation. Thus, nonclonotypic TCRVβ targeting broadens our perspectives on human T cell response modes and might offer ways to induce clinically beneficial phenotypes in defined T cell subsets.

## Teaser

Triggering αβ T cell receptors via germline regions induces a distinct phenotype with potential utility in cancer immunotherapy.

## INTRODUCTION

Differential mobilization of functionally distinct T lymphocyte subsets, including cytolytic, regulatory, proinflammatory, and follicular helper cells, contributes substantially to variations in T cell–mediated responses to infections and cancer and to variable propensity to autoimmune disease (*1*, *2*). In addition, αβ T cell subsets can display varying temporospatial states, including short-term effectors and a spectrum of durable memory cells including tissue-resident memory (T_RM_) cells (*3*, *4*), supplementing which T cells can show different states of responsiveness, that include functionally competent, quiescent, senescent, anergic, and exhausted (*5*–*7*). Understanding the basis for these states informs us of T cell biology and offers ways to better understand immune deficiencies and immunopathologies and to improve T cell therapeutics.

Although T cell heterogeneity and status are regulated by multiple inputs, including costimulatory, coinhibitory, and cytokine receptors (*8*, *9*), how T cells respond to stimulation is fundamentally influenced by the status of T cell receptor (TCR) activation. For example, during T cell development, self-antigens binding the TCR with relatively high affinity can induce death, anergy, or immunoregulatory phenotypes, whereas those bound with lower affinity can induce positive selection (*10*). Moreover, whether peripheral T cells will mature as purely short-lived effectors, acquire durable systemic or T_RM_ including stem-like “resource” cells, or differentiate toward exhaustion is influenced by variations in the affinity, avidity and chronicity of TCR engagements with antigenic cargoes presented by major histocompatibility complex (MHC), CD1, or MR1 (*11*). Such clonotypic variation is an underpinning foundation of adaptive immunity and is a target for manipulation in attempts to improve T cell–mediated immunotherapies.

However, the TCR can also act as an innate receptor, engaging ligands via nonclonal, germline-encoded sites (*12*). Thus, so-called superantigen (SAg) proteins of microbes and endogenous viruses commonly engage TCRVβ sequences encoded in CDR2 and hypervariable region 4 (HV4) (*13*). SAg interactions can have qualitatively distinct consequences from clonotypic, CDR3-mediated interactions, but interpretations of this are complicated by coengagement of CDR3 motifs, at least in the cases of Streptococcal Pyogenes Enterotoxin C (SpeC) and Staphylococcal Enterotoxin H (SEH) (*14*, *15*), and by SAg binding to MHC class II proteins that may variably interact with additional points on the TCR (*16*–*18*).

By contrast, we recently showed that subtypes of mouse and human γδ TCRs use germline-encoded Vγ CDR2 and HV4 regions for nonclonotypic engagements of endogenous butyrophilin-like (BTNL) proteins without any obvious involvement of CDR3 or MHC (*19*, *20*). Those engagements induced distinct phenotypic changes, including proliferation, differentiation, and improved cell survival that collectively evoke positive T cell selection, consistent with which BTNL-dependent TCR activation events are essential for the normal development of discrete tissue-associated γδ T cell compartments defined by Vγ chain usage (*21*–*23*). These data raise the question of whether innate nonclonotypic engagement of germline TCR V-region residues might likewise induce distinct phenotypes in αβ T cells (*12*).

While not precluding their existence, there are no known self-encoded equivalents of BTNL ligands for αβ TCRs. Therefore, we used antibodies specific for discrete human Vβ regions. Given the number of human Vβ regions and their relatedness, we hypothesized that the high specificity of several such anti-Vβ antibodies might rely on their engaging regions close to or within CDR2 and HV4, akin to BTNL binding to Vγ. Here, we use mutations and structural biology to show that this hypothesis is correct for several Vβ-specific reagents. Moreover, the anti-Vβ reagents most commonly induced polyclonal human T cells to adopt distinct, memory-like effector (“T_MLE_”) phenotypes unlike other commonly described human T cell states including those induced by anti-CD3 or SAg stimulation.

These findings expose a qualitatively distinct modality by which the TCR might influence T cell status in development, infection, autoimmune disease, and cancer. Moreover, in therapeutic settings, nonclonotypic Vβ-dependent stimulation might facilitate the selective activation and/or maintenance of defined subcompartments of the αβ T cell repertoire, thereby limiting massive cytokine release, activation-induced death, and exhaustion of residual T cells that are common consequences of pan-αβ T cell activation by agonist anti-CD3 antibodies and immune checkpoint inhibition (*24*). Furthermore, anti-Vβ reagents may permit selective regulation of defined Vβ-subsets with clinically important disease associations (*25*, *26*).

## RESULTS

### Antibody binding to germline-encoded regions of TRBV

To investigate the impact of engaging germline-encoded human TCRVβ regions, we used a series of humanized antibodies specific for the proteins encoded by *TRBV6-5* (also known as Vβ13.1), *TRBV5-1* (also known as Vβ5.1), *TRBV12-3/4* (also known as Vβ8), and *TRBV20-1* (also known as Vβ2), respectively, and for each of which the Fc region was mutated (N297A), thereby limiting Fc Receptor (FcR) engagement.

**Fig. 1.**
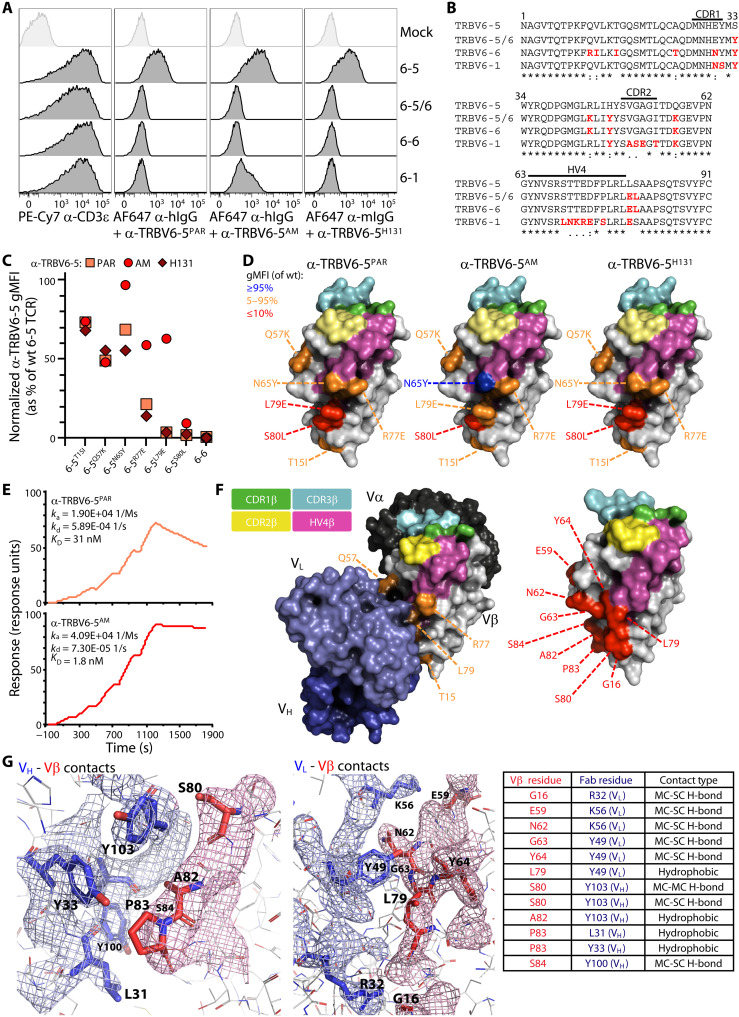
Mapping of amino acids involved in the binding of α-TRBV antibodies. (**A**) Flow cytometry analysis of TCR staining on J76 cells coexpressing a fixed TCRα chain (TRAV24) and TCRβ chains bearing the indicated TRBV segments. Cells were stained separately with the indicated antibodies 72 hours posttransduction. TRBV6-5/6 is a chimeric construct derived from TRBV6-5 and TRBV6-6. Representative of three independent transductions. (**B**) Alignment of V regions (leader and CDR3 excluded) of the TCRβ constructs stained in (A). Amino acids in red indicate differences from TRBV6-5. (**C**) Summary flow cytometry analysis of the staining of the indicated TRBV6-5 mutants and TRBV6-6 negative control. MFI normalized to α-CD3ɛ and expressed as % of the wild-type (wt) TRBV6-5 staining. Representative of three independent transductions. (**D**) Mapping of the amino acids mutated in (C). The Vβ domain structure was derived from Protein Data Bank (PDB) 6JXR. The CDR1, CDR2, CDR3, and HV4 regions are highlighted in green, yellow, cyan and magenta, respectively. (**E**) Single-cycle kinetics analysis of the binding of the α-TRBV6-5^PAR^ (top) and α-TRBV6-5^AM^ (bottom) antibodies to a TRAV12-3/TRBV6-5 αβ TCR [sequence derived from PDB 4WWK (*68*)] by surface plasmon resonance. (**F**) Left: Crystal structure of the α-TRBV6-5^AM^ antibody bound to a TRAV12-3/TRBV6-5 αβ TCR [sequence derived from PDB 4WWK (*68*)]. Color key for the Vβ annotation as previously, including mutations shown to affect the staining of a TRBV6-5 TCR expressed by J76 cells [see (D)] by the α-TRBV6-5^PAR^ antibody. Light blue, V_L_; dark blue, V_H_. Right: Vβ domain amino acids determined to contact the antibody are highlighted in red (see below). (**G**) Detailed view of the contacts between the α-TRBV6-5^AM^ antibody V_H_ (left) and V_L_ (middle) domains and the Vβ domain amino acids. Mesh structures represent electron densities at interfaces. Pairings are listed as a table (right).

To understand how the antibodies engaged Vβ, flow cytometry was used to measure the reactivity of anti–TRBV6-5 antibodies to TCR-deficient J76 cells (derived from Jurkat cells) that had been cotransduced with constructs encoding, respectively, a fixed TCRα chain [TRAV24, clone RA14 (*27*)] and the indicated TCRβ chains: TRBV6-5, TRBV6-1, TRBV6-6, and a chimaera of TRBV6-5 and TRBV6-6 (TRBV6-5/6). Anti-CD3 staining indicated comparable expression of these TCRs on transduced J76 cells (Fig. 1A, left). The antibodies used were a mouse anti–TRBV6-5 originally derived by Kappler and colleagues (*28*) (clone H131, here referred to as “anti–TRBV6-5^H131^”), a humanized version of H131 (“anti–TRBV6-5^PAR^”), and an affinity-matured version of anti–TRBV6-5^PAR^ (“anti–TRBV6-5^AM^”) [for details on affinity maturation, see Materials and Methods]. Each antibody clearly detected TRBV6-5^+^ J76 cells, but neither TRBV6-6^+^ J76 cells nor cells transduced with the TRBV6-5/6 chimaera (Fig. 1A). Affinity maturation was associated with increased cross-reactivity toward TRBV6-1^+^ J76 cells (Fig. 1A).

Correlating antibody reactivities to protein sequence alignments (Fig. 1B), we hypothesized that binding might require amino acids within or close to germline-encoded CDR2 and/or HV4, akin to where BTNL proteins bind Vγ chains (*19*, *20*). To test this, we assessed reactivity against J76 cells transduced with TRBV6-5 mutants. Germane to this, we noted in parallel that anti-CD3 reactivity of primary human T cells was decreased on cells engaging anti–TRBV6-5: note median fluorescence intensity (MFI) for anti-CD3ɛ (*x* axis) for TRBV6-5^+^ cells (blue) versus TRBV6-5^NEG^ cells (black) (fig. S1A). This was likewise true for the other anti-TRBV antibodies (fig. S1A, bottom). This may be explained by steric hindrance of anti-CD3ɛ antibody (OKT3) binding, given that CD3ɛ is positioned relatively close to Vβ (fig. S1B). Preincubating J76 cells expressing different Vβ chains led to a dose-dependent decrease in anti-CD3ɛ staining (fig. S1C). For this reason, anti-TRBV staining of J76 cells expressing mutant TCRs was quantified after normalization to anti-CD3 staining performed in parallel (Fig. 1C and fig. S1D).

This analysis identified residues critical to binding [e.g., S80 (red)], together with residues that impaired but did not abrogate reactivity [e.g., Q57 (orange)] and residues with negligible or no impact (depicted as blue) (Fig. 1, C and D, and fig. S1D). Expectedly, some mutations had less impact on anti–TRBV6-5^AM^ (e.g., L79E) or had no impact at all (N65Y) (Fig. 1, C and D, and fig. S1D). Alignment of residues partially or completely impairing binding with Fig. 1B shows that they were commonly proximal to or within CDR2 (amino acids 49 to 55) or HV4 (amino acids 64 to 79), which are depicted on Fig. 1D as yellow (CDR2) and magenta (HV4) versus CDR1 (green) and CDR3 (cyan).

Essentially, the same held true for the humanized antibodies against TRBV5-1, TRBV12-3/4, and TRBV20-1 and for the mouse antibodies from which those derived (fig. S1, E and F). Of note, for anti–TRBV5-1, there was critical dependence on two residues, T53 and S70, that are central to CDR2 and HV4, respectively, whereas the reactivity of anti–TRBV20-1 was unaffected by mutation of two residues, A72 and L76, that are both central to HV4 (fig. S1, E and F) but was affected by L68 which is within HV4. In sum, although their fine specificities were distinct, the reactivity of each anti-TRBV reagent was commonly determined fully or in part by germline-encoded residues within or juxtaposing CDR2 and HV4.

Surface plasmon resonance confirmed binding of anti–TRBV6-5^PAR^ to a TCR comprising TRBV6-5 and TRAV12-3 and demonstrated the higher affinity of anti–TRBV6-5^AM^ (Fig. 1E). We then deduced the crystal structure of α-TRBV6-5^AM^ bound to a TRAV12-3/TRBV6-5 TCR and in so doing identified contact residues (Fig. 1F and table S1) largely consistent with the mapping data described above. Thus, except for G16, which is encoded within FR1, all evident contact residues (Fig. 1F, red) were encoded between amino acids 59 and 64 and between 79 and 84 (Fig. 1, F and G). Given that no residue between 59 and 64 is different between TRBV6-5 and TRBV6-6, the failure of anti–TRBV6-5 to detect TRBV6-6 is probably centrally explained by engagement of L79 and S80, which in TRBV6-6 are E and L, respectively (see Fig. 1B). L79 makes hydrophobic interactions with the V_L_ chain, while S80 displays main chain–focused and main-chain–side-chain interactions with V_H_ together with electron densities (Fig. 1G). In sum, the Vβ-specific antibodies tested here offered a means to engage αβ TCRs via germline-encoded Vβ sequences approximately aligned to Vγ residues engaging BTNL proteins.

### Biological sequelae of anti-TRBV binding

**Fig. 2.**
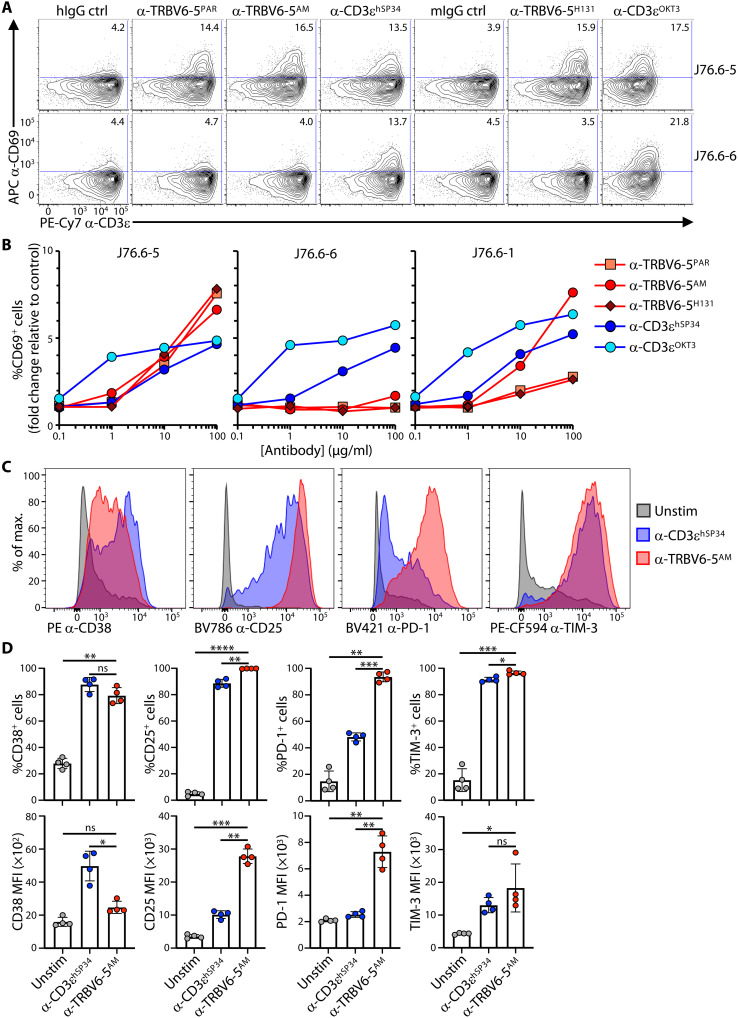
Stimulatory activity of α-TRBV antibodies. (**A**) J76 cells coexpressing a TRAV24^+^ TCRα chain and a TRBV6-5^+^ (J76.6-5) or TRBV6-6^+^ (J76.6-6) TCRβ chain were seeded on the indicated plate-bound antibodies (10 μg/ml), incubated for 4 hours at 37°C, and then stained for analysis of CD69 up-regulation by flow cytometry. Representative of two independent experiments. (**B**) J76 cells coexpressing a TRAV24^+^ TCRα chain and a TRBV6-5^+^ (J76.6-5), TRBV6-6^+^ (J76.6-6), or TRBV6-1^+^ TCRβ chain were incubated for 4 hours at 37°C with increasing concentrations of the indicated plate-bound antibodies and then stained for analysis of CD69 up-regulation by flow cytometry. Representative of two independent experiments. (**C** and **D**) Purified T cells were cultured for 7 days without (Unstim) or with the indicated plate-bound antibodies in complete media supplemented with IL-2 and IL-15 and then stained for the indicated activation markers for analysis by flow cytometry after gating on TRBV6-5^+^ cells. Representative histograms and summary data of the percentage of positive cells and MFI for *n* = 4 donors are shown in (C) and (D), respectively. In (D), data are shown as mean ± SD. ns, not significant; *P < 0.05; **P < 0.01; ***P < 0.001; ****P < 0.0001 [paired analysis of variance (ANOVA) with Dunnett’s method, comparing α-TRBV6-5^AM^ to every other condition individually].

Because human γδ TCR engagement by BTNL molecules induces CD69 expression on J76 cells transduced with cognate γδ TCRs, we investigated whether anti-Vβ antibodies did likewise. When compared to either a humanized, FcR-disabled anti-CD3ɛ (α-CD3ɛ^hSP34^), commonly used in bispecific T cell engager therapeutics, or the widely used human CD3ɛ-specific mouse antibody clone OKT3 (α-CD3ɛ^OKT3^), plate-bound anti–TRBV6-5 antibodies induced CD69 within 4 hours specifically on J76 cells transduced with TRBV6-5–containing TCRs but not TRBV6-6–containing TCRs (Fig. 2A). This biological outcome measure was concentration dependent and, for anti–TRBV6-5^AM^, showed some cross-reactivity for TRBV6-1–expressing cells (Fig. 2B), consistent with epitope mapping described above.

Essentially equivalent results were obtained with all other anti-TRBV antibodies tested (fig. S2, A to C), and again those anti-TRBV antibodies showed no notable activation of J76 cells expressing closely related but noncognate Vβ regions: e.g., TRBV5-4 for anti–TRBV5-1, TRBV12-5 for anti–TRBV12-3/12-4, and TRBV29-1 for anti–TRBV20-1 (Fig. 2B and fig. S2, A to C). Usually, OKT3 was more active at lower concentrations of antibody, but at higher concentrations, anti-TRBV reagents activated cells to comparable or higher levels (Fig. 2B and fig. S2, A to C).

These data provided the platform to examine how primary T cells might respond to sustained exposure to antibodies specific for germline-encoded Vβ sequences. Thus, purified human T lymphocytes were cultured for 7 days on plate-bound antibodies, using concentrations based on the comparable capacities of anti–TRBV6-5^AM^ and anti-CD3ɛ^hSP34^ to induce CD69 (above). For every condition, changes in cell surface phenotype were assessed on cells gated on the cognate TRBV-regions (e.g., TRBV6-5^+^ cells reactive to anti–TRBV6-5). As before, illustrative flow plots are shown together with multidonor summaries.

Each of the four anti-TRBV antibodies up-regulated the frequency of cells expressing CD38, a widely used marker of human T cell activation (Fig. 2, C and D, and fig. S2D), although the levels of expression per cell (MFI) were significantly weaker than with anti-CD3ɛ^hSP34^ (used as a comparator) (Fig. 2, C and D). By contrast, anti–TRBV6-5^AM^ induced conspicuously uniform and very high CD25 up-regulation compared to anti-CD3ɛ^hSP34^, with significantly increased MFI (Fig. 2, C and D). Note that the affinities of the two antibodies are comparable (*K*_D_ = 3 nM for anti-CD3ɛ^hSP34^; *K*_D_ = 1.8 nM for anti–TRBV6-5^AM^), so differences in affinity are an unlikely basis for the differences in CD38 and CD25 induction. Of note, other anti-TRBV antibodies conspicuously phenocopied the impacts of anti–TRBV6-5^AM^ (fig. S2D).

PD-1 and TIM-3, which are inhibitory receptors up-regulated by robust TCR stimulation, were also uniformly up-regulated by anti-TRBV antibodies, with significantly greater increases in PD-1 clearly distinguishing anti–TRBV6-5^AM^ from anti-CD3ɛ^hSP34^ (Fig. 2, C and D, and fig. S2D). Of note, PD-1 up-regulation was an acute activation response to anti-TRBV engagement, being induced on ~60% of total CD25^+^ cells after only 72 hours, with this fraction becoming ~90% by 120 hours (day 5) [fig. S2, E (top) and F]. When gating on TRVB6-5^+^ cells, ~100% expressed PD-1 by the 120-hour time point [fig. S2, E (bottom) and F], suggesting that residual CD25^+^PD-1^NEG^ cells may have been those expressing TCRs (e.g., TRBV6-1) that respond less strongly to anti–TRBV6-5^AM^ engagement. In sum, TRBV-specific antibodies induced a signature activation phenotype, as assessed by CD25, CD38, and PD-1 expression levels, that was displayed by the great majority of cognate T cells and that was significantly different from the collective phenotypes of αβ T cells responding to anti-CD3ɛ.

### Anti-TRBV reagents induce highly proliferative T cells

**Fig. 3.**
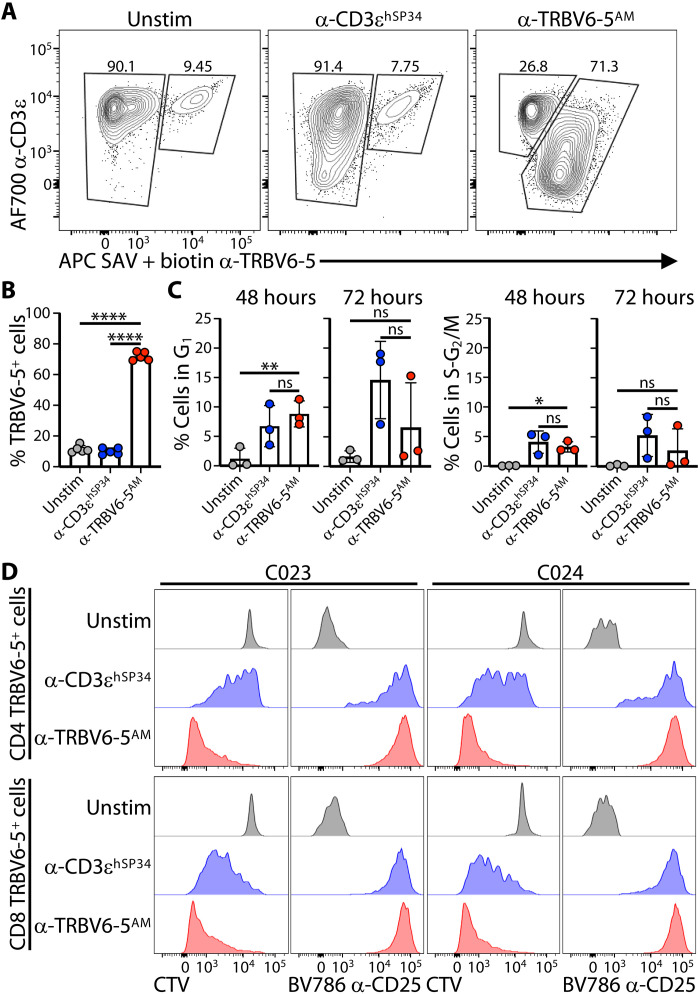
α-TRBV–induced T cell proliferation. (**A** and **B**) Purified T cells were cultured as in Fig. 1 (C and D) without (Unstim) or with the indicated plate-bound antibodies and then analyzed by flow cytometry to evaluate the specific enrichment for TRBV6-5^+^ cells by the corresponding affinity-matured (AM) antibody. Representative contour plots and summary data of the percentage of TRBV6-5^+^ cells for *n* = 5 donors are shown in (A) and (B), respectively. In (B), data are shown as mean ± SD. ****P < 0.0001 (paired ANOVA with Dunnett’s method, comparing α-TRBV6-5^AM^ to every other condition individually). (**C**) Purified T cells were cultured without (Unstim) or with the indicated antibodies in the presence of IL-2 and IL-15 and then stained intracellularly for Ki67 and DNA content (Hoescht 33342) at 48 and 72 hours poststimulation to determine the percentage of cells in G_1_ (left) and S/G_2_M (right). Data are shown as the mean ± SD of *n* = 3 donors. *P < 0.05; **P < 0.01 (paired ANOVA with Dunnett’s method, comparing α-TRBV6-5^AM^ to every other condition individually). Representative contour plots for one donor are shown in fig. S3B. (**D**) Purified T cells were labeled with cell trace violet (CTV), then cultured for 5 days in the presence of IL-2 + IL-15 without (Unstim) or with the indicated plate-bound antibodies, and then stained for CD25 for analysis of activation and proliferation (CTV dilution) of CD4 and CD8 cells by flow cytometry. Representative of 10 different donors.

When stimulated with plate-bound anti-TRBV antibodies, the cultures became dominated by expanding cells expressing the cognate TRBV regions, whereas although such cells were also activated in anti-CD3–stimulated cultures, there was, as anticipated, no change in their frequency relative to T cells expressing other TCRs (Fig. 3. A and B, and fig. S3A). Note that the enrichment for TRBV6-5–expressing cells was greater for anti–TRBV6-5^PAR^ than for anti–TRBV6-5^AM^, consistent with the latter showing cross-reactivities to cells bearing TCRs composed of TRBV6-1, TRBV6-2, and TRBV6-3 regions and to the closely related TRBV10-3, but not to TRBV6-6 which indeed showed no enrichment (fig. S3A). In addition, there was no evidence for clonal expansions of TRBV6-5^+^ cells in response to anti–TRBV6-5, i.e., no clones were being disproportionately selected for. Illustrating this, usage of all 13 J gene segments (*TRBJ1-6*; *TRBJ2-7*) recombined to TRBV6-5 gene segments remained largely comparable over 11 days in anti–TRBV6-5^AM^–stimulated cultures relative to unstimulated cultures or to those cultured with anti-CD3ɛ^OKT3^ (fig. S3B).

The representative flow plots (Fig. 3A) illustrated that the MFIs for CD3ɛ and for TRBV6-5 were greatly down-regulated on the expanded cells cultured with anti–TRBV6-5. Whereas it might be argued that this could reflect some masking of Vβ6-5 detection by small amounts of detached plate-bound anti–TRBV6-5, this was minimized by our use of a biotinylated form of anti–TRBV6-5 that was in turn detected with streptavidin (Fig. 3A). Rather, TCR down-regulation is an established consequence of active TCR-transduced signaling, in which regard it was noteworthy that down-regulation with anti–TRBV6-5^AM^ was stronger than with anti-CD3e^hSP34^ cultures, which seems provocatively similar to very strong TCR down-regulation shown by BTNL-stimulated human γδ T cells (*22*).

To test whether the increased representation of cognate TRBV^+^ cells primarily reflected cell cycling, as opposed to death of other cells, we used a recently developed flow cytometry protocol using Hoechst, Ki67, and relaxed size-based cell gating to accurately assess the fractions of cells in G_1_ and S-G_2_/M, respectively (fig. S3C). Exposure to either anti–TRBV6-5^AM^ or anti-CD3ɛ^hSP34^ for 48 hours induced significant and comparable (six- to sevenfold) increases in cells in G_1_, relative to unstimulated cultures sustained in interleukin-2 (IL-2) and IL-15 (Fig. 3C and fig. S3C). Notwithstanding high inter-individual variation in the response, there were also increased proportions of cells in S-G_2_/M (Fig. 3C and fig. S3C). By 72 hours, the percentages of cycling cells had declined somewhat in anti-TRBV–stimulated cultures, but frequencies remained mostly well above those in unstimulated cultures (Fig. 3C and fig. S3C). Note that at any one time point, the percentages of cells in S-G_2_/M will be low even in highly proliferating cultures. Therefore, the collective impact on cell cycling of anti-TRBV was also assessed by examining cell tracer violet (CTV) dilution over 5-day cultures.

**Fig. 4.**
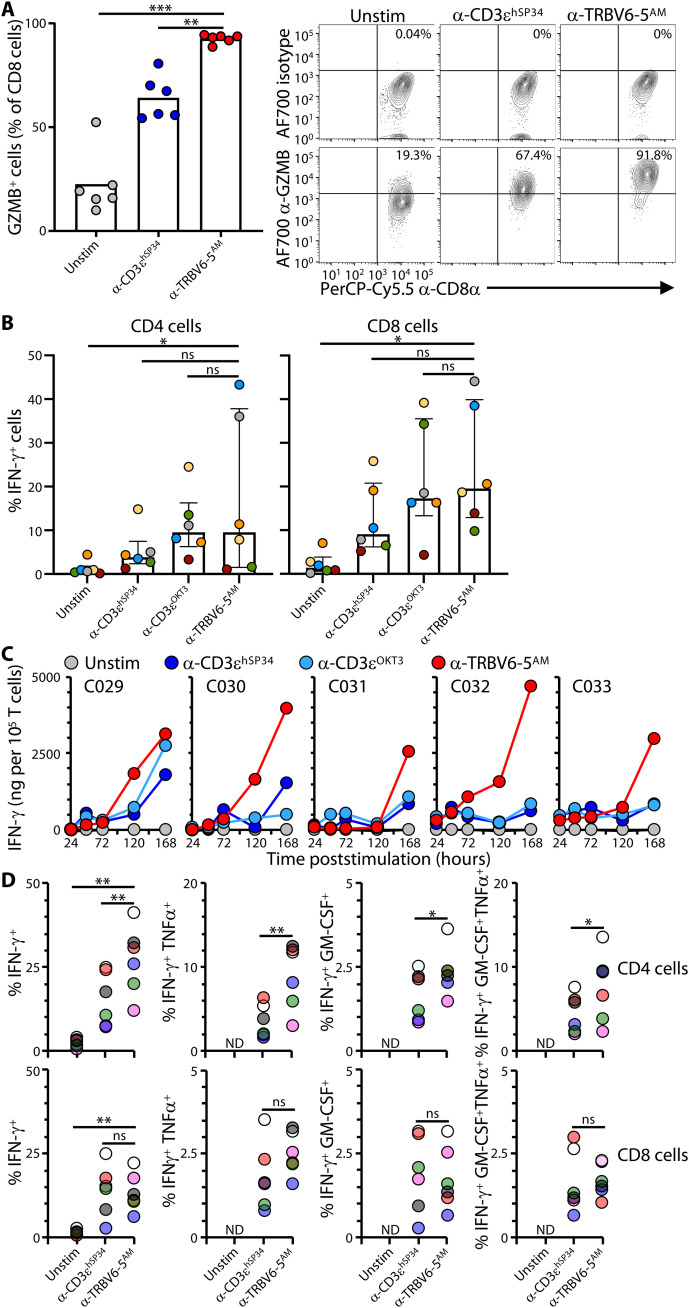
α-TRBV–induced effector responses. (**A**) Purified T cells were cultured as in Fig. 2 (C and D) without (Unstim) or with the indicated plate-bound antibodies for 7 days then stained intracellularly for GZMB or a matching isotype control for analysis by flow cytometry. Data are shown as mean ± SD of *n* = 6 donors. **P < 0.01; ***P < 0.001 (paired ANOVA with Dunnett’s method, comparing α-TRBV6-5^AM^ to every other condition individually). Representative contour plots for one donor are shown on the right. (**B**) Purified T cells were cultured as in Fig. 2 (C and D) without (Unstim) or with the indicated plate-bound antibodies for 3, 5, and 7 days and then stained intracellularly for IFN-γ or a matching isotype control for analysis by flow cytometry in CD4 (top row) and CD8 (bottom row) cells. Data are shown as mean ± SD of *n* = 6 donors. *P < 0.05 (paired ANOVA with Dunnett’s method, comparing α-TRBV6-5^AM^ to every other condition individually). Summary data for all time points are shown in fig. S4A. (**C**) Purified T cells from *n* = 5 donors were cultured as in Fig. 2 (C and D) without (Unstim) or with the indicated plate-bound antibodies, and supernatants were collected at the indicated time points to evaluate the cumulative secretion of IFN-γ by flow cytometry using the T_H_ response and antiviral response LEGENDplex assays. (**D**) Purified T cells were cultured as in Fig. 2 (C and D) without (Unstim) or with the indicated plate-bound antibodies for 7 days and then stained intracellularly for IFN-γ, GM-CSF, and TNFα for analysis by flow cytometry in CD4 (top row) and CD8 (bottom row) cells. Each color represents a different donor (*n* = 6). ND, not determined; *P < 0.05; **P < 0.01 (paired ANOVA with Dunnett’s method, comparing α-TRBV6-5^AM^ to every other condition individually for IFN-γ^+^ cells; paired *t* test for cells producing multiple cytokines).

With every cell division, CTV dilution occurs stepwise by ~50%, and by assessing this incremental progression in anti-CD3ɛ–stimulated cultures (Fig. 3D), we could estimate that there were ~6 to 7 divisions over the 5-day period (Fig. 3D). Notably, almost all TRBV6-5^+^ cells exposed to anti–TRBV6-5^AM^ had fully diluted their CTV after 5 days (Fig. 3D, showing data for two donors; see fig. S3D for illustrative plots). Thus, we could conclude that most such cells would have been proliferating over the full extent of the culture period. The cells were likewise uniformly CD25^+^, with both traits applying equally to CD4^+^ and CD8^+^ cells (Fig. 3D and fig. S3D). CD4^+^ and CD8^+^ TRBV6-5^+^ cells showed variable CTV dilution after 5 days in culture with α-CD3ɛ^hSP34^ (Fig. 4D), but this was anticipated given that they had to compete with all other T cells receiving stimulation via anti-CD3ɛ. In sum, cells targeted by anti-TRBV reagents were uniformly activated (CD25^+^) and highly proliferative, contemporaneous with their near-uniform expression of the inhibitory receptors, PD-1 and TIM-3.

### Anti-TRBV reagents induce high effector potentials

The heightened activation state of the anti-TRBV–stimulated cells was further demonstrated by effector potentials. Thus, essentially, all CD8 T cells stimulated with plate-bound anti–TRBV6-5^AM^ up-regulated granzyme B (GZMB) by day 7 (Fig. 4A) and given that the cells were not gated on TRBV6-5 expression, the GZMB^NEG^ cells were most likely residual non–TRBV6-5^+^ cells: Note that because of TCR down-regulation (above), it is challenging to accurately segregate highly stimulated TRBV6-5^+^ cells from bona fide TRBV6-5^NEG^ cells. By contrast, GZMB up-regulation was observed in a significantly lower percentage of αβ T cells stimulated with anti-CD3ɛ^hSP34^ (Fig. 4A).

Likewise, when total CD4 and CD8 T cells were examined following 7-day cultures, the frequencies of cells producing interferon-γ (IFN-γ) protein induced by anti–TRBV6-5^AM^ were, on average, comparable with or higher than frequencies induced by either anti-CD3ɛ^hSP34^ or anti-CD3ɛ^OKT3^ notwithstanding inter-individual variation (Fig. 4B; to facilitate comparisons, colored dots identify donors; left hand graphs; compare yellow and green donors for which anti-CD3ɛ^OKT3^ gave greater stimulation, with gray and blue dots, for which anti–TRBV6-5^AM^ gave greater stimulation). Note that this strong effector cytokine up-regulation was observed without restimulation in vitro. When examined kinetically, ~10% of total CD8 T cells had become IFN-γ^+^ after only 72-hour culture with anti–TRBV6-5^AM^ at a time when the cognate Vβ^+^ cells typically accounted for less than 25% of the CD8 T cells (fig. S4A). The frequency of IFN-γ–expressing CD8^+^ and CD4^+^ T cells peaked by day 5 (120 hours) and was mostly maintained after 7 days (168 hours), particularly for CD8 T cells (fig. S4A).

When the supernatants of total T cells cultured with anti–TRBV6-5^AM^ and anti-CD3ɛ antibodies were harvested over time, the amounts of IFN-γ produced continued to rise substantially by day 7 for every donor and, for most, were conspicuously higher than the amounts detected in anti-CD3ɛ–stimulated cultures (Fig. 4C and table S2). The fact that IFN-γ was not clearly detected in any cultures until day 5 presumably reflects a low sensitivity of the assay by comparison to intracellular staining.

IL-4, IL-17A, IL-10, IL-6, and IP-10 were either undetected or detected at much lower levels and showed greater donor-to-donor variation [fig. S4, B and C; compare *y*-axis scales with Fig. 4C ; table S2]. However, some tumor necrosis factor–α (TNFα) and granulocyte-macrophage colony-stimulating factor (GM-CSF) were detected (table S2), and we therefore gated on IFN-γ–producing CD4^+^ and CD8^+^ T cells poststimulation with either anti–TRBV6-5^AM^ or anti-CD3ɛ^hSP34^ to determine whether there were IFN-γ,GM-CSF and IFN-γ,TNFα double producing cells and likewise IFN-γ,GM-CSF,TNFα triple producers (fig. S4D). Polycytokine producers occurred at higher frequencies among anti–TRBV6-5^AM^ versus anti-CD3ɛ^hSP34^–stimulated CD4 T cells, with TNFα the more commonly coexpressed cytokine, and they occurred at comparable frequencies among CD8 T cells stimulated by anti–TRBV6-5^AM^ versus anti-CD3ɛ^hSP34^ (Fig. 4D). Thus, anti-TRBV–stimulated cells can display a notable T helper cell 1 (T_H_1)/T cytolytic 1 (Tc1) effector response, although this skewing may have been partly influenced by culturing cells in IL-2 and IL-15.

Correlating with the T_H_1/Tc1 response of cells to anti–TRBV6-5^AM^ was expression of T-BET protein, as visualized by intracellular flow cytometry (fig. S4, E and F). Not only did a significantly greater frequency of CD8 T cells express T-BET following anti–TRBV6-5^AM^ versus anti-CD3ɛ^hSP34^ stimulation, but the level of expression per cell (captured by MFI) was significantly greater for both CD8 T cells and CD4 T cells responding to anti–TRBV6-5^AM^ (fig. S4E). By contrast, there was no induction of FoxP3 that is associated with a regulatory T cell (T_reg_) fate (*29*), with some suggestion that the relative frequency of FoxP3^+^ cells de facto decreased among CD4 T cells responding to anti–TRBV6-5^AM^ (fig. S4, E and F). In sum, anti–TRBV6-5^AM^ induced strong effector differentiation across responding CD4 and CD8 T cells that, together with the strong proliferative response considered above, attest to a heightened state of activation. Moreover, wherever it was examined, other anti-TRBV antibodies induced comparable phenotypic outcomes, which we next sought to examine in greater detail.

### Anti-TRBV stimulation induces a signature T_MEM_ phenotype

By contrast to primary T cells in mice that are mostly naïve before any manipulation, human T cells at steady state are variably heterogeneous (*30*) and routinely classified using surface markers CD45RA and CCR7 as naive (T_N_; CD4RA^+^CCR7^+^), central memory (T_CM_; CD4RA^−^CCR7^+^), effector memory (T_EM_; CD4RA^−^CCR7^−^), and effector memory reexpressing CD45RA (T_EMRA_; CD4RA^+^CCR7) (*31*, *32*). We therefore asked whether sustained exposure to anti-TRBV reagents might affect these states.

**Fig. 5.**
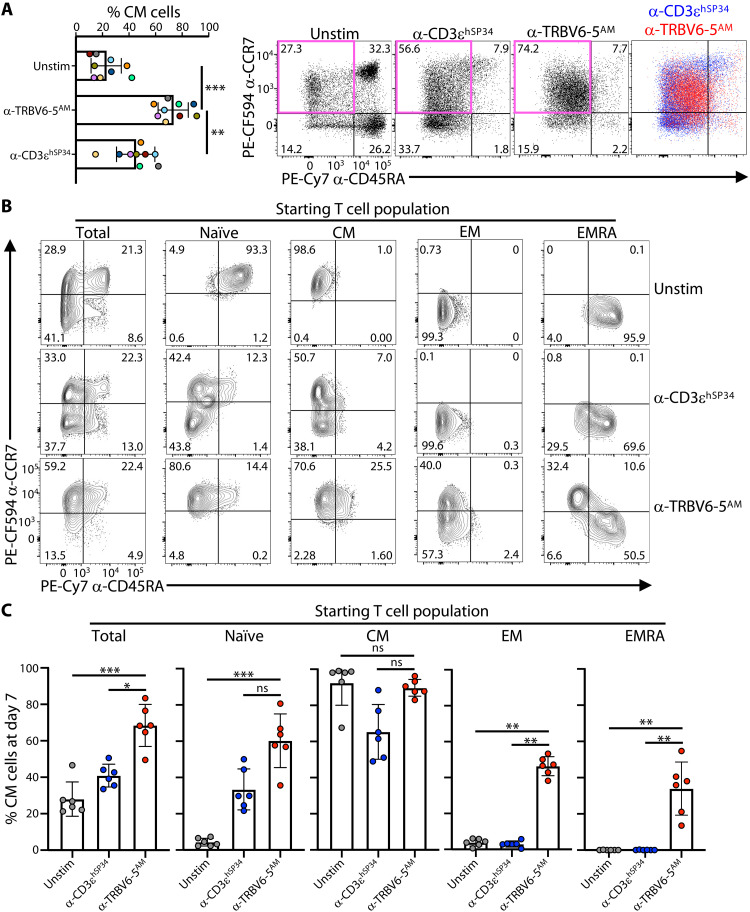
α-TRBV antibodies promote a “CM-like” phenotype. (**A**) Purified T cells were cultured for 7 days without (Unstim) or with the indicated plate-bound antibodies as in Fig. 2 (C and D) and then stained for CD45RA and CCR7 to determine the proportion of naïve and memory phenotypes by flow cytometry. Summary data are shown for the resulting percentages of CD45RA^NEG^CCR7^+^ central memory (CM) cells for *n* = 9 donors, as mean ± SD. **P < 0.01; ***P < 0.001 (paired ANOVA with Dunnett’s method, comparing α-TRBV6-5^AM^ to every other condition individually). Example contour plots for one donor are provided (right-hand) with T_CM_ gate delineated in pink. (**B** and **C**) Purified T cells or the indicated presorted subsets were cultured for 7 days without (Unstim) or with the indicated plate-bound antibodies as in Fig. 2 (C and D) and then stained for CD45RA and CCR7 to determine the proportion of naïve and memory phenotypes by flow cytometry. Representative contour plots for one donor and summary data of the resulting proportion of CM cells for *n* = 6 donors are shown in (B) and (C), respectively. In (C), data are shown as mean ± SD. *P < 0.05; **P < 0.01; ***P < 0.001 (paired ANOVA with Dunnett’s method, comparing α-TRBV6-5^AM^ to every other condition individually).

Examining 7-day cultures of purified primary T cells from each of nine independent donors, we found that anti–TRBV6-5^AM^ versus anti-CD3ɛ^hSP34^ consistently induced higher frequencies of cells with a “T_CM_-like” profile (Fig. 5A; note that colors identify individual donors, facilitating direct cross-comparisons of treatments): Such cells accounted for 59 to 95% of all T cells (Fig. 5, A and B, left), a distribution range largely non-overlapping with anti-CD3ɛ^hSP34^–stimulated cells. When the impacts of anti–TRBV6-5^AM^ versus anti-CD3ɛ^hSP34^ were directly compared (Fig. 5A, bottom), it was also evident that the positioning of the cells in the T_CM_ quadrant was slightly different, with lower CCR7 expression and higher CD45RA expression for anti–TRBV6-5^AM^–stimulated (red) versus anti-CD3ɛ^hSP34^–stimulated (blue) cells. For this reason, we initially adopted the term, T_CM_-like for the phenotype acquired by most anti–TRBV6-5^AM^–stimulated cells, an issue that is returned to below.

To investigate the origin of the T_CM_-like cells, we next purified starting populations of T_N_, T_CM_, T_EM_, and T_EMRA_ cells from different donors and cocultured those with anti–TRBV6-5^AM^. In every case, there was a notable conversion toward the T_CM_-like phenotype (Fig. 5. B and C), which was unexpected for T_EM_ and T_EMRA_ cells, and although this was seldom sufficient to become the dominant phenotype when starting with T_EM_ or T_EMRA_ cells, it contrasted notably with the complete failure of anti-CD3ɛ^hSP34^ to drive any such conversion: Anti-CD3ɛ^hSP34^ converted a substantial fraction of purified T_CM_ cells toward T_EM_ (Fig. 5, B and C). Thus, the defining markers of T_EM_ and T_EMRA_ cells were reverted by the cells’ exposure to anti–TRBV6-5^AM^, arguing that Vβ stimulation may have a generalized potential to revert cells from different end stages.

Further exploring the origins of the T_CM_-like phenotype, we considered the highly proliferative state of anti–TRBV6-5–stimulated cells (described above), particularly given that Ki67 expression, which is a hallmark of actively cycling cells, is reportedly a signature of T_CM_ cells (*33*). Thus, we exposed human T cells for 5 days to anti-CD3ɛ^hSP34^ and then subdivided the cells into four categories in order of increased proliferative activation, CTV^HI^CD25^NEG^, CTV^HI^CD25^+^, CTV^MED^CD25^+^, and CTV^LO^CD25^+^ and then assessing the status of each subset using CD45RA and CCR7. For both CD4 and CD8 T cells from three independent donors, the cells with increased proliferation showed increased skewing toward the T_CM_ phenotype at the expense of the T_EM_ phenotype (fig. S5). Thus, proliferative capacity is associated with memory T cell status, and hence, the relatively high proportion of T_CM_-like cells induced by anti-TRBV stimulation might in part relate to their high degree of proliferation.

Notwithstanding high proliferation being associated with higher proportions of T_CM_ cells, the signature, near-uniform pattern of CD45 and CCR7 staining of anti-TRBV–stimulated cells within the T_CM_ quadrant (see above) prompted us to hypothesize that anti–TRBV6-5–induced T_CM_-like cells might be defined by unique combinations of traits when compared to conventional T_CM_ cells and to other major human T cell phenotypes described to date. To test this, we assessed the cells’ phenotypes in more detail, complementing single-cell resolution by flow cytometry with transcriptomics.

### Single-cell transcriptomics of anti-TRBV versus anti-CD3ɛ–stimulated cells

**Fig. 6.**
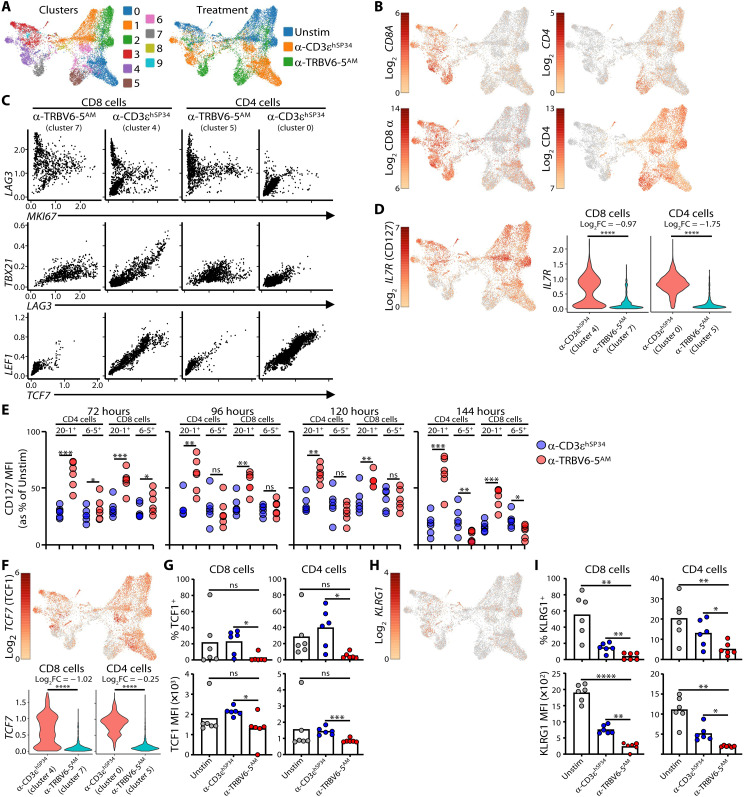
Single-cell transcriptome analysis α-TRBV–stimulated cells. (**A** and **B**) Purified T cells were cultured for 7 days with the indicated plate-bound antibodies or rested overnight without cytokines (Unstim) and then processed for single-cell RNA sequencing analysis. UMAP representation of clusters and treatment groups are shown in (A); CD4 and CD8 cells are identified in (B) using gene expression (top row) and barcoded antibodies (bottom row). (**C**) Imputed counts of the indicated genes were used in scatterplots to visualize coexpression patterns across clusters and cell types. (**D**) UMAP representation of *IL7R* expression (left) and violin plots (imputed expression, right). Log_2_FC, log2 fold change; ****P < 0.0001 [Wilcoxon rank sum test with Bonferroni correction, from the differentially expressed gene (DEG) analysis]. (**E**) Purified T cells were cultured without (Unstim) or with the indicated plate-bound antibodies and cell-surface expression of CD127 on the indicated subsets was monitored by flow cytometry at the indicated time points (*n* = 6 donors). *P < 0.05; **P < 0.01; ***P < 0.001 (paired *t* test). (**F**) UMAP representation of *TCF7* expression (top row) and violin plots (imputed expression, bottom row). ****P < 0.0001 (Wilcoxon rank sum test with Bonferroni correction, from the DEG analysis). (**G**) Purified T cells were cultured for 7 days without (Unstim) or with the indicated plate-bound antibodies and intracellular TCF1 expression was measured in CD4 or CD8 cells. (**H**) UMAP representation of *KLRG1* expression. (**I**) Purified T cells were cultured for 7 days without (Unstim) or with the indicated plate-bound antibodies and cell surface KLRG1 expression was measured on CD4 or CD8 cells. Data in (G) and (I) are the mean (bars) of percentage positive cells (top row) and MFI of positive cells (bottom row) for *n* = 6 donors. *P < 0.05; **P < 0.01; ***P < 0.001; ****P < 0.0001 (paired ANOVA with Dunnett’s method, comparing α-TRBV6-5^AM^ to every other condition individually).

Purified live T cells from three independent donors were collected after 7 days in culture with either plate-bound anti-CD3ɛ^hSP34^ or plate-bound anti–TRBV6-5^AM^, or after overnight culture with complete media without cytokines (“Unstim”) and were then subjected to single-cell analyses to evaluate transcript levels (single-cell RNA sequencing) and protein abundance [cellular indexing of transcriptomes and epitopes (CITE-Seq)], using 29 hash-tagged antibodies (see Materials and Methods)]. Data were processed and normalized, and protein abundance was used to guide the spatial distribution and clustering of cells (Fig. 6A, left), onto which were mapped the three experimental conditions (Fig. 6A, right) and the expression levels of CD4 and CD8α transcripts (Fig. 6B, top) and proteins (Fig. 6B, bottom).

De facto, clusters will constitute groupings of heterogeneous cells, and TRBV6-5^AM^ will not induce a wholly uniform phenotype. For these reasons, it was notable that most CD8^+^ T cells activated by plate-bound anti–TRBV6-5^AM^ were found within a single cluster 7 [Fig. 6, A (gray) and B), and most CD4^+^ T cells grouped together in cluster 5 [Fig. 6, A (brown) and B], whereas anti-CD3ɛ^hSP34^–stimulated CD8^+^ T cells were mostly found in cluster 4 (Fig. 6A, purple), and anti-CD3ɛ^hSP34^–stimulated CD4^+^ T cells were grouped mostly in cluster 0 (Fig. 6A, blue), with a small percentage in cluster 6 (Fig. 6A, pink). TRBV6-5 transcripts were elevated in cluster 7 and cluster 5, as expected for cells activated by anti–TRBV6-5 (table S2), and, consistent with the antibody cross-reactivity noted above, TRBV6-1 transcripts were also enriched in CD4 T cells stimulated by anti–TRBV6-5^AM^ (table S2). The segregation of CD8 and CD4 T cells activated by anti–TRBV6-5^AM^ and anti-CD3ɛ^hSP34^, respectively, into different and distinct clusters clearly offers prima facie support for the hypothesis that the two modes of TCR engagement induce unique consensus states.

The gene expression patterns in clusters 7 and 5 corroborated key findings (above) for cells stimulated by anti–TRBV6-5^AM^. Thus, most cells expressed *MKI67* (encodes Ki67), indicative of cycling and proliferation and, for CD8 T cells *MKI67* expression, was higher than for anti-CD3ɛ^hSP34^–stimulated CD8^+^ cells (cluster 4) (fig. S6A, top and bottom). Likewise, anti–TRBV6-5^AM^–stimulated cells expressed *IL2RA* (encodes CD25), *GZMB*, *HAVCR2* (encodes TIM-3), and *LAG3* that encodes an additional inhibitory marker of activated T cells, and in each case, expression levels in CD8 cells exceeded those in CD3ɛ^hSP34^-stimulated cells CD8 cells, with the same being true for *HAVCR2* and *LAG3* in CD4^+^ T cells (fig. S6A, top and bottom, and table S3).

Although *LAG3* and *MKI67* expression were correlated for many cells stimulated by anti-CD3ɛ^hSP34^ or anti–TRBV6-5^AM^, there were noticeably few *LAG3*^LO^*MKI67*^LO^ cells following anti–TRBV6-5^AM^ stimulation (clusters 7 and 5), whereas this was the modal state for anti-CD3ɛ^hSP34^–stimulated cells (clusters 4 and 0) (Fig. 6C). Clusters 7, 5, and 4 also contained *LAG3*^HI^ cells with negligible *MKI67* expression, consistent with some highly activated cells exiting the cell cycle (Fig. 6C).

*LAG3* was also commonly coexpressed with *TBX21* encoding T-BET protein, which was detected in anti–TRBV6-5^AM^–stimulated and anti-CD3ɛ^hSP34^–stimulated cells (above). Again, however, very few anti–TRBV6-5^AM^–stimulated cells expressed neither *LAG3* nor *TBX21* (cluster 7 and cluster 5), whereas this was commonly true for anti-CD3ɛ^hSP34^–stimulated cells (Fig. 6C). In sum, anti–TRBV6-5^AM^–stimulated cells could be distinguished from anti-CD3ɛ^hSP34^–stimulated cells by their coexpression patterns of several genes associated with proliferative and effector activation and/or activation-induced inhibitory receptors.

Reciprocally, anti–TRBV6-5^AM^–stimulated cluster 7 and cluster 5 cells could frequently be distinguished by their near-uniform failure to express several other genes associated with discrete T cell states. For example, *IL7R* (encodes CD127) was strongly expressed by unstimulated T cells [e.g., compare Fig. 6D (left) with Fig. 6A (right)], but its expression was almost completely down-regulated in anti–TRBV6-5^AM^–stimulated T cells (Fig. 6D). Investigating this at the protein level, we performed a time course, observing down-regulation beginning within the first 72 hours of stimulation. We compared the specific response of Vβ6-5^+^ cells with the response of Vβ20-1^+^ cells to which anti–TRBV6-5^AM^ does not cross-react. As expected, CD127 down-regulation was most evident on Vβ6-5^+^ cells, but there was over time some down-regulation on Vβ20-1^+^ cells, indicating the existence of a bystander suppression of CD127 (Fig. 6E and fig. S6B), which might explain why the initial down-regulation in anti-CD3–stimulated cultures was so rapid (Fig. 6E and fig. S6B). Nonetheless, down-regulation by 144 hours was significantly greater in Vβ6-5^+^ cells stimulated with anti–TRBV6-5^AM^ versus anti-CD3, consistent with the transcriptomic data (Fig. 6D).

*TCF7* (encodes TCF1) and *KLRG1* transcripts were also expressed at conspicuously low levels, and it was confirmed by flow cytometry that very few anti–TRBV6-5^AM^–stimulated cells expressed the respective proteins, by comparison to their expression by ≥20% (TCF1) and > 10% (KLRG1) of anti-CD3ɛ^hSP34^–stimulated cells [Fig. 6, F to I: Note that *KLRG1* transcript counts were so low as to make quantitative differential expression estimates unreliable; fig. S6C: Note that the quadrant lines were determined by fluorescence minus one (FMO) plots, and differences across conditions and between CD4 versus CD8 cells reflect differences in cell morphology, size, etc. in each treatment group].

In several types of naïve and memory T cells, TCF1 expression is closely correlated with the expression levels of LEF1 and KLF2 (*34*), as was reflected in *TCF7* and *LEF1* coexpression by anti-CD3ɛ^hSP34^–stimulated cluster 4 and cluster 0 cells. Most anti–TRBV6-5^AM^–stimulated cluster 7 and cluster 5 cells were negative for both *TCF7* and *LEF1* (Fig. 6C) and also lacked *KLF2* (fig. S6D). Likewise, TCF1 expression is correlated with BCL6 (*35*, *36*) expression, and jointly, they suppress expression of BLIMP1, encoded by *PRDM1* (*37*), reflected in a mutually exclusive pattern of *PRDM1* and *BCL6* in many cluster 4 CD8^+^ T cells (fig. S6, E and F). By contrast, this mutually exclusive relationship was overtly lost in clusters 7 and 5, in which *BCL6* seemed comparably expressed by cells across a full range of *PRDM1* expression (fig. S6, E and F).

### Anti-TRBV–stimulated cells and memory T cell differentiation

Having obtained single-cell transcriptomic differentiation of cells stimulated by anti–TRBV6-5^AM^ versus anti-CD3ɛ, we sought more insight into the precise phenotypes of anti–TRBV6-5^AM^–stimulated cells. First, our pro tem classification of the cells as T_CM_-like based on near-uniform CCR7^+^CD45RA^NEG/LO^ staining (above) was challenged by the lack of *IL7R* and *TCF7*, which are usually expressed by T_CM_ cells, combined with high expression of T-BET, IFN-γ, and GZMB that usually are not expressed by T_CM_ cells. Whereas those parameters might collectively suggest a recently activated effector phenotype, an effector classification is confounded by lack of KLRG1 expression. Thus, because KLRG1^NEG^ cells are reportedly the source of all memory T cell types (*38*), we hypothesized that anti-TRBV stimulation induced a KLRG1^NEG^IL7R^NEG^ phenotype that in mice reportedly identifies cells transitioning from effectors to memory precursors (*39*). Hence, from this point on, we considered anti-TRBV–stimulated cells as effectors with memory potential, i.e., T_MLE_ cells, and investigated them further by assessing their expression of transcription factors associated with memory T cell differentiation.

To better understand the signature phenotype induced by anti–TRBV6-5^AM^, we first segregated anti–TRBV6-5^AM^–stimulated cells by expression of RNAs encoding TRBV6-5, TRBV6-1, TRBV6-2, and TRBV10-3 (“targeted” TRBVs) versus “nontargeted” cells expressing RNAs for other defined TRBVs. Note that this is preferable to comparing with TRBV6-5^NEG^ cells because it avoids mistakenly applying the term TRBV6-5^NEG^ to cells TRBV6-5^+^ cells that display activation-induced TCR down-regulation or that were drop-outs for TRBV segment sequencing. The datasets (table S4) are illustrated for the differential expression of genes encoding transcription factors and related molecules associated with memory T cell differentiation (fig. S7A).

**Fig. 7.**
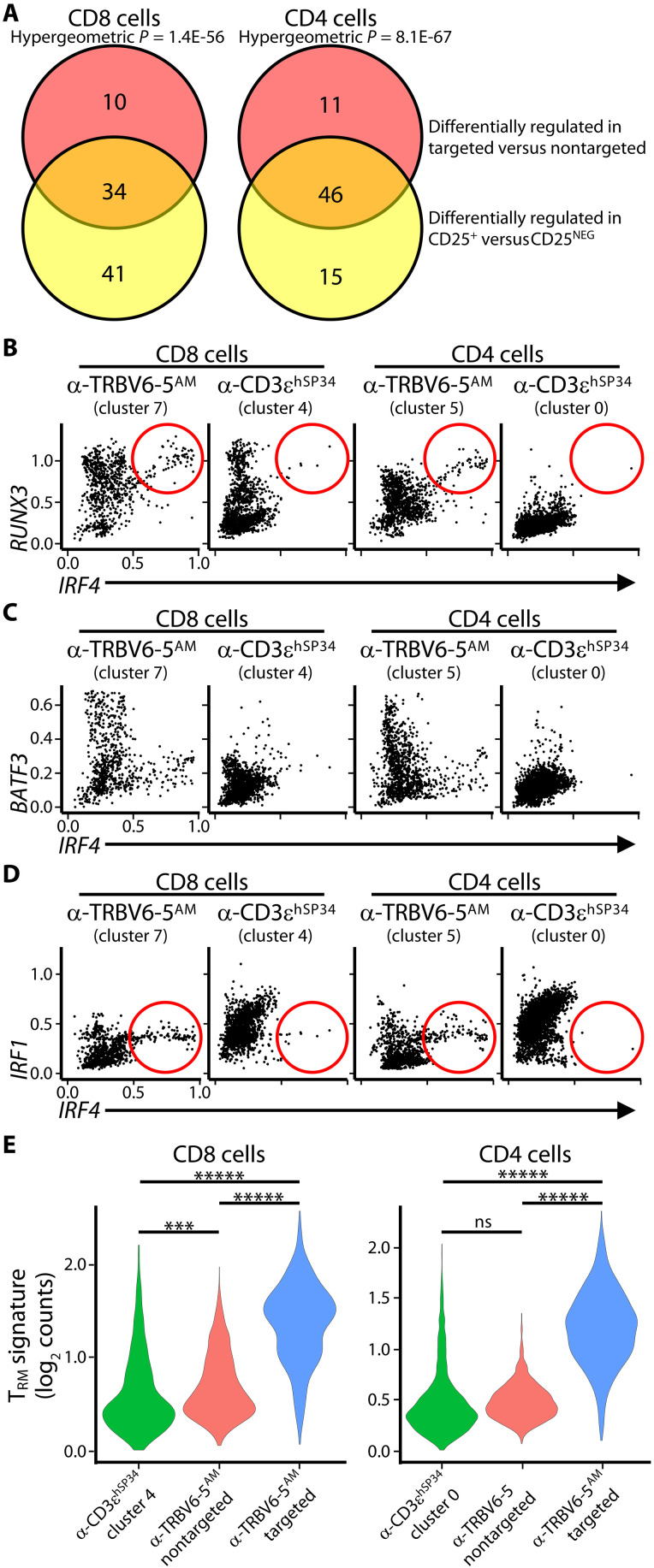
Transcription factor profile and validation of α-TRBV stimulated cells. (**A**) Venn diagrams showing the number of transcription factors (TFs) that are uniquely and commonly up/down-regulated across groups. Only TFs that were significantly differentially expressed were included. Significance calculated using hypergeometric test. (**B** to **D**) Scatterplots with the imputed expression of genes highlighted, divided by cell type and treatment groups. (**E**) Violin plots representing the overall expression of genes in the T_RM_ gene signature. The *y* axis indicates the log_2_ of the raw counts. ***P < 0.001; *****P < 2.22 × 10^−16^.

Second, we segregated anti–TRBV6-5^AM^–stimulated cells by *IL2RA* expression as a discriminator of activated versus non-activated cells within the same cultures, the validation of which was our finding that CD25^+^ versus CD25^NEG^ CD4^+^ and CD8^+^ T cells were enriched in TRBV6-5 transcripts (table S5). There were conspicuous overlaps of transcription factor genes differentially expressed by targeted TRBV-expressing cells and by CD25^+^ cells, as illustrated for CD8^+^ T cells (fig. S7B), and in aggregate, these overlaps were highly significant (Fig. 7A). For example, both datasets confirmed the substantial relative loss of *TCF7* and *LEF1* expression, described above (fig. S7, A and B, and tables S4 and S5). In addition, to validate the single-cell RNA sequencing data, we subjected anti–TRBV6-5^AM^–stimulated cells of several independent donors to NanoString analyses of selected gene panels (table S6), and further consideration (below) is given only to those differentially expressed, memory-associated genes that were unequivocally validated by NanoString and/or flow cytometry.

We first examined *RUNX3*, which encodes a chromatin remodeling factor that facilitates effector T cell differentiation, particularly for CD8^+^ T cells, and that skews cells toward memory states, thereby limiting their terminal differentiation (*40*). Its expression has been associated with GZMB and IFN-γ (*41*), both expressed by anti–TRBV6-5^AM^–stimulated cells, and it reportedly cooperates with BLIMP1 (*PRDM1*) and IRF4. *RUNX3*, *PRDM1*, and *IRF4* transcripts were all significantly up-regulated in targeted TRBV^+^ and CD25^+^ cells [fig. S7, A and B (gray arrows) and tables S4 and S5], with IRF4 RNA and protein more significantly induced in anti–TRBV6-5^AM^–stimulated than in anti-CD3ɛ^hSP34^–stimulated cells (fig. S7, C and E). Moreover, anti–TRBV6-5^AM^–stimulated cells included *IRF4*^HI^ cells that near-uniformly coexpressed *RUNX3*, whereas this was rarely observed for anti-CD3ɛ^hSP34^–stimulated cluster 4 and cluster 0 cells (Fig. 7B, circled).

Second, we focused on *BATF3* which encodes a chromatin remodeling protein that also cooperates with IRF proteins (*42*) and that promotes CD8^+^ T cell survival and memory differentiation, to the extent that it has been proposed to optimize cells used in cancer immunotherapy (*43*). TRBV-targeted and CD25^+^ CD8^+^ T cells showed strong *BATF3* induction (fig. S7, A and B, green arrows), whereas *BATF3*^HI^ cells were essentially absent from anti-CD3ɛ^hSP34^–stimulated cluster 4 and cluster 0 cells (Fig. 7C).

Third, we revisited IRF factors. In complete contrast to most anti-CD3ɛ^hSP34^–stimulated cells, there was strong down-regulation of *IRF1* [fig. S7, A and B (black arrows) and tables S4 and S5], which encodes a protein that also can collaborate with BATF factors, e.g., in inducing IL-10–producing T cells (*44*). This would seem consistent with anti-TRBV stimulation skewing cells away from some discrete effector phenotypes (e.g., inducible T_reg_ cells) and toward others (e.g., the observed Tc1/T_H_1 bias), although this should again be considered in the context of the maintenance cytokines used. Provocatively, however, some cells coexpressed *IRF1* and *IRF4*, whereas these were very rare among anti-CD3ɛ^hSP34^–stimulated clusters (Fig. 7D, circled).

IRF4, which was strongly up-regulated in many anti–TRBV6-5^AM^–stimulated cells (above), reportedly facilitates aerobic glycolysis (*45*), including via up-regulation of SLC2A3 (*46*), a glucose transporter, together with SLC3A2, an amino acid transporter that has also been shown to be essential for activated CD8^+^ T cell expansion (*47*). Of note, *SLC2A3* was up-regulated in all CD25^+^ and TRBV-targeted T cells, and *SLC3A2* was up-regulated in CD25^+^ and targeted CD8^+^ T cells (tables S4 and S5). In sum, anti–TRBV6-5^AM^–stimulated cells were characterized by several key markers of activation and memory differentiation, including mediators of high energy consumption, but the coexpression pattern of some such markers were atypical by comparison to current frames of reference.

Nonetheless, we next considered the status of the cells in terms of exhaustion, anergy, quiescence, or senescence. IRF4 has been associated with terminally exhausted T cells that can coexpress PD-1, LAG3, TIM-3, and *PRDM1* and lack TCF1 (*48*), an expression pattern that described a substantial fraction of anti-TRBV–stimulated cells. However, an exhausted state is improbable given the complete absence of *TOX* induction when assessed by any of our primary or validation assays and given the high proliferative and effector states of anti-TRBV–stimulated cells described above. Those data likewise argue against the cells being anergic, and in the same vein, it seems implausible that the T cells are either senescent since they lack KLRG1, or quiescent, since they lack KLF2 (above).

Last, because RUNX3 has also been implicated in T_RM_ differentiation (*49*), we examined whether there were hints of progression toward extralymphoid T cells. Indicative of this, activated cells from anti–TRBV6-5^AM^–stimulated cultures showed notable induction of *BHLHE40* (fig. S7, A and B, orange arrows) encoding a factor regulating the mitochondrial fitness and epigenetic stability of T_RM_ and tumor-infiltrating T cells (*50*). We therefore assessed a documented T_RM_-associated gene signature (see Materials and Methods) for its expression by CD4^+^ and CD8^+^ T cells that were anti-CD3ɛ^hSP34^ stimulated or that were anti–TRBV6-5^AM^ stimulated and segregated into targeted TRBV^+^ cells versus nontargeted cells. Although they do not express a full T_RM_ signature, consistent with their lymphoid circulation and CCR7 expression, targeted anti–TRBV6-5^AM^–stimulated cells showed highly significant enrichments for T_RM_ signature genes relative to nontargeted cells or to T cells stimulated with anti-CD3ɛ^hSP34^ (Fig. 7E). These similarities in gene expression to T_RM_ further support our decision to apply the term T_MLE_ as an aggregate descriptor of the cells’ collective traits.

### SAgs and anti-Vβ antibodies induce distinct phenotypes

**Fig. 8.**
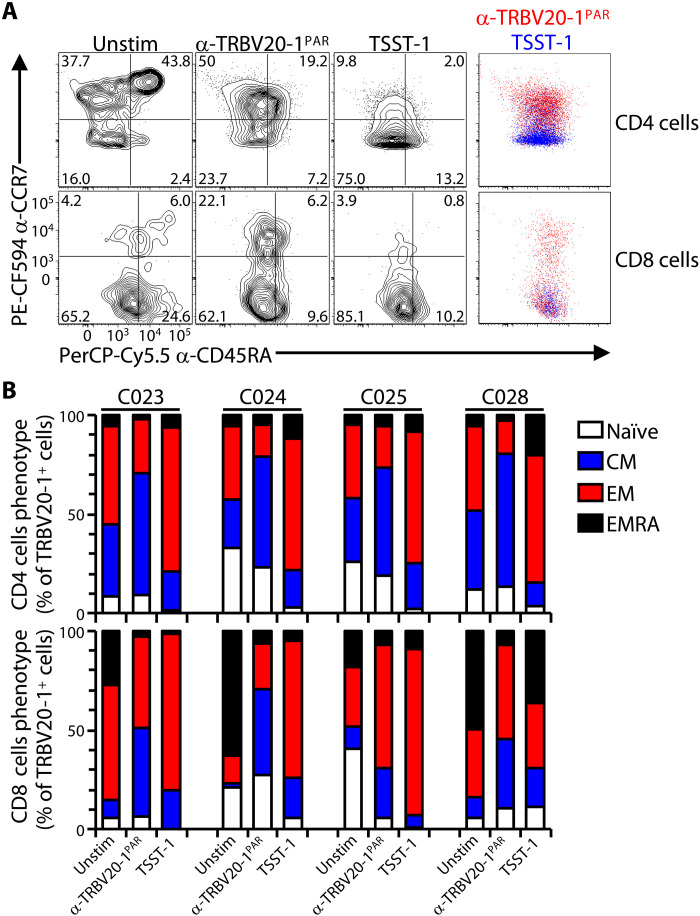
Distinct responses to α-TRBV antibodies versus SAgs. (**A**) Purified PBMC were cultured for 7 days without (Unstim) or with plate-bound α-TRBV20-1^PAR^ antibody or TSST-1 as in Fig. 2 (C and D) and then stained for CD45RA and CCR7 to analyze the resulting memory phenotypes by flow cytometry after gating on TRBV20-1^+^ cells, for both CD4 (top row) and CD8 (bottom row) cells. Representative data for one donor (C025) for each subset and condition (contour plots) and overlays of the α-TRBV20-1^PAR^ versus TSST-1 conditions (colored dot plots, right) are shown. (**B**) Summary data of the proportion of each naïve and memory subsets in the indicated conditions for *n* = 4 donors.

The engagement of germline-encoded domains of Vβ regions finds precedent in the action of microbial SAgs (*13*). We therefore asked whether the consequences of anti-TRBV engagements were phenocopies of those induced by SAgs. Among those TRBV regions targeted by the antibodies used in this study, TRBV20-1^+^ (a.k.a. Vβ2^+^) cells are targeted by the SAg toxin shock syndrom toxin 1 (TSST-1) (*51*, *52*), which competes with anti–TRBV20-1^PAR^ (but not anti–TRBV6-5^PAR^) for staining αβ T cells (fig. S8A). For these assays, purified T cells could not be used because notwithstanding MHC class II up-regulation on activated T cells, SAgs are customarily presented by myeloid cells constitutively expressing MHC class II (*53*). Therefore, peripheral blood mononuclear cells (PBMCs) were stimulated in parallel with either TSST-1 or anti–TRBV20-1^PAR^. The illustrative data clearly show that TSST-1 primarily drove CD4^+^ TRBV20-1^+^ T cells toward T_EM_, compared to the T_CM_-like phenotypes driven by anti–TRBV20-1^PAR^ (Fig. 8A), and this pattern was consistent across four independent donors (Fig. 8B).

The starting populations of CD8^+^ T cells for three of four donors were markedly enriched in T_EM_ or T_EMRA_ cells, perhaps a sequel to the severe acute respiratory syndrome coronavirus 2 pandemic which arose during this study (Fig. 8, A and B). Nonetheless, reprising the plasticity studies described above, anti–TRBV20-1^PAR^ treatment conspicuously increased the ratio of CD45RA^NEG^CCR7^+^ cells to T_CM_ cells, whereas TSST-1 treatment did not do this, instead favoring T_EM_/T_EMRA_ (Fig. 8, A and B, and fig. S8B). Thus, the outcomes of SAg and anti-TRBV stimulation were very different, despite considerable overlap of their epitopes on TCR-Vβ (fig. S8C).

## DISCUSSION

Perspectives on TCR biology have been broadened by the finding of natural physiologic, repertoire-selecting ligands for mouse and human TCR Vγ chains. By virtue of their nonclonotypic interactions with germline-encoded residues, these interactions are, by definition innate, and their consequences for γδ cells include several sequelae that collectively equate to positive selection (*12*). Inevitably, this raises a question of whether engagement of germline TCR V-region residues might likewise evoke distinct phenotypes in αβ T cells. Although SAg stimulation can engage nonclonal regions, primarily encoded by TRBV segments, those interactions can be complicated by variable involvements of CDR3 motifs and the concurrent binding of SAgs to MHC class II proteins which may have further interactions with the TCR (*13*).

Therefore, to explore the consequences of engaging human αβ TCRs only via germline-encoded regions, we have as a proof of principle used TRBV-specific antibodies that we have demonstrated by genetics and biochemistry to engage residues within or close to the counterpart Vγ regions engaged by BTNL molecules, namely, CDR2 and HV4 (*19*, *20*). We have found that the outcomes for selectively activated subsets of primary human T cells, either purified or in the context of PBMCs, were profoundly different to outcomes induced in primary T cells either by anti-CD3 (the most used TCR agonist) or by SAgs. Moreover, the outcomes seem distinct from other commonly described phenotypes of human T cells responding to MHC-restricted antigens in a multiplicity of settings, and hence, we have proposed a new term, T_MLE_, for this phenotype.

Predictably, anti-TRBV stimulation of primary human T cells did not induce a single homogeneous outcome, and moreover, a potential limitation of the study is the variable, donor-to-donor status of the starting T cells, with conspicuously higher resting levels of T_EM_ and T_EMRA_ being evident after COVID-19 (*54*). Another limitation is that much of the study focuses on the responses of cells to one type of reagent, namely, TRBV6-5 antibodies, either parental or affinity matured, although the traits induced were largely phenocopied by other anti-TRBV reagents wherever this was examined. Last, we also concede that we examined a limited set of time points following stimulation by a limited set of antibody concentrations.

These issues notwithstanding, primary T cell cultures from all donors examined responded to anti-TRBV by displaying phenotypes that were near-uniform by several criteria, including CD25 up-regulation, implying hyperresponsiveness to IL-2; induction of other markers of acute T cell stimulation, including CD69, GZMB, and the inhibitory receptors PD-1, TIM-3, and LAG3; negligible expression of IL7R, TCF1, KLF2, LEF, and KLRG1; and the cells’ focusing on an unusual expression pattern of CD45RA and CCR7 within the “T_CM_” gate. Albeit that it was not considered in detail here, there was also strong induction of costimulatory molecules, including OX40 and ICOS, that render the cells amenable to further activation via heterotypic cell-cell interactions.

Whereas the T cell phenotype was particularly clearly induced in naïve T cells, it was also the case that anti–TRBV6-5 stimulation could revert purified T_EM_ cells and T_EMRA_ cells to a CD45RA^NEG^CCR7^+^ phenotype, whereas anti-CD3ɛ^hSP34^ entirely failed to do this. Thus, anti–TRBV6-5 stimulation exposed a hitherto understudied plasticity of T_EM_ and T_EMRA_ cells that may be potentially useful in understanding T cells biology and in promoting T cell renewal. This plasticity may contribute to the functional intra-clonal heterogeneity shown by T cells following different infectious challenges or vaccines (*8*).

At the core of the phenotype induced by anti–TRBV6-5 is a set of coexpression and coexclusion patterns for genes and gene products implicated in T cell differentiation and in T cell status, which distinguishes anti–TRBV6-5–stimulated cells from those stimulated by anti-CD3, by SAgs, and by other agonists. The high degrees of coexpression and coexclusion coupled with a quasi-uniform phenotype defined by flow cytometry and single-cell transcriptomics counter the argument that anti–TRBV6-5–stimulated cells comprise a mixture that is so heterogeneous as to confound alignment with previously described αβ T cell subsets. Thus, we conclude that TRBV engagement promotes in cognate T cells an induction and expansion of T_MLE_ phenotypes rarely described hitherto.

Several of the consequences of TRBV engagement suggest that the cells are transitioning through a highly activated state to one or more effector-competent memory T cell states, including a putative potential to establish tissue residency. Among the traits underpinning this conclusion are the cells’ multiple rounds of proliferation; their high expression of cytokines and granzymes, their failure to express KLRG1, IL7R, TCF1, *LEF1*, and *KLF2*; and their overt up-regulation of *RUNX3*, *BATF3*, and IRF4 (note that all such observations were validated by more than one independent technique). Moreover, the cells’ broad expression of inhibitory receptors, e.g., PD-1, LAG3, and TIM-3, may be viewed in the context of inhibitory signaling being a sentinel of strong agonist activation and of it sustaining a distinct early memory CD8 T cell precursor that is resistant to DNA damage (*55*), i.e., the cells’ status is maintained by engagement of stromal ligands. The induction of a memory precursor would also be consistent with the KLRG1^NEG^IL7R^NEG^ phenotype describing murine cells transitioning from effectors to memory precursors (*39*). Conversely, there was no clear basis for considering the cells as exhausted. We also note that many such aspects of TRBV stimulation applied comparably to CD4 and CD8 T cells, whereas before this, RUNX3 and several other factors have been primarily viewed in the context of CD8^+^ T cell differentiation (*56*).

Prima facie, these outcomes have some parallels with BTNL-mediated, Vγ-dependent activation of γδ TCRs that at early times of development induces targeted cells to proliferate and establish stable residency in extralymphoid niches, wherein they share with memory T cells a capacity for rapid effector responsiveness, enhanced by signaling from innate receptors, e.g., NKG2D (*21*–*23*, *57*). It therefore seems reasonable to speculate that nonclonotypic signals received selectively via innate TRBV motifs, albeit via as-yet unidentified self or microbial ligands, might contribute to shaping complex human germline TRBV repertoires (*12*, *58*). Although BTNL engagement does not induce significant increases in effector functions (GZMB and IFN-γ) (*22*) as is seen for anti-TRBV–stimulated αβ T cells, it does strongly skew cells toward a Tc1/T_H_1 phenotype when the cells are subsequently activated (*59*, *60*). Blood Vγ9Vδ2 T cells, which are evidently regulated by nonclonal interactions with BTN2A1 (*61*, *62*), show some intriguing phenotypic similarities to anti-TRBV–stimulated αβ T cells, including their signature pattern of CD45RA and CCR7/CD27 expression (*63*).

The prospect that engaging different sites on the TCR might induce different outcomes is an intriguing one. Although precedent was to some extent established by SAgs, their induction of distinct phenotypes versus anti-CD3 stimulation is complicated by the additional involvement of MHC class II. By contrast, we remain unclear as to what underpins the stark differences between cells activated by anti-TRBV versus anti-CD3, the latter commonly considered as the paradigm of nonclonotypic TCR signaling. Physical influences on signal transduction were demonstrated by evidence for differential depending on the orientation TCRαβ adopts vis-à-vis peptide-MHC (pMHC) (*64*). Conceivably too, the differential response might be related to the marked TCR down-regulation induced by anti-TRBV which is akin to that provoked by TCRγδ engagement of BTNL3 + BTNL8 (*22*). In addition, the impact of Vβ engagement via CDR2 + HV4 might be transduced to an FG loop at the Vβ-Cβ interface that amplifies TCR signaling in response to force (*65*). Understanding these unexpected differences should be a subject of future studies.

In sum, engaging TCRs via germline-encoded subregions has, under the conditions used, reproducibly induced an aggregate T_MLE_ phenotype that can expand our perspectives of T cell biology. Future studies may determine whether there are natural circumstances in which αβ T_MLE_ cells are detectable, and if so, the nature of their inducing agent(s). Meantime, the distinct outcomes of TRBV stimulation strongly suggest the utility of this approach for manipulating T cells in vitro and in vivo in clinical settings, particularly cancer immunotherapy. As was recently considered (*24*), the strategy of stimulating only a defined subset of αβ T cells (e.g., TRBV6-5^+^) might simultaneously reduce the deleterious clinical impacts of pan-T cell stimulation using anti-CD3. Furthermore, the approach might promote the beneficial activation of Vβ-specific T cell subsets associated with responses to discrete immunological challenges (*25*, *26*).

## MATERIALS AND METHODS

### Study design

The study was conducted to ascertain the outcome of stimulating human T cells via germline-encoded regions of TCRVβ by comparison to the use of anti-CD3, a universally used modality for stimulating T cells. The data were collected from the investigation of primary human T cells obtained from widely available blood cones. The methods used were those established in the academic community as appropriate measures of T cell responsiveness and were wide-ranging. To test the hypothesis that at least some parameters of T cell activation would be significantly different for cells stimulated with anti-TCRVβ reagents versus anti-CD3. This remained the hypothesis under test throughout.

#### Sample size

We did not use a formal power calculation because in a fundamental research study of this form, we had no concept of whether the main experimental comparator groups would differ at all, let alone by what magnitude. Once the pilot experiments had been completed, we used sufficient samples to permit us to achieve statistical significance for quantitative changes of >20% in key parameters, e.g., surface protein expression, cells producing cytokines, and cells in defined stages of the cell cycle. Of note, achieving power without an excessive sample number requires protocol optimizations that reduce technical (as opposed to biological) coefficients of variation, and hence, considerable efforts were expended in achieving this.

#### Data collection, inclusion/exclusion criteria, and outliers

We limited data collection when we had ascertained which parameters displayed highly significant quantitative differences or qualitative differences. Thus, we do not deny that some criteria for which differences between groups were not significant might have shown differences if much higher sample numbers were used, but such putative differences are not central to the hypothesis under test. No data were excluded where the experiment fulfilled the quality control for the optimized procedures. If outliers existed, they are presented in the data.

#### Selection of endpoints

Endpoints were prospectively selected, and the appropriate statistical corrections made, based on an extensive literature, including our own.

#### Replicates

The numbers of replicates are indicated in each figure panel and legend.

#### Research subjects or units of investigation

All PBMC samples were isolated from commercially available, anonymized human peripheral blood samples (a.k.a. cones) from healthy donors, provided by the NHS Blood and Transplant Service.

#### Experimental design

This study was performed under controlled laboratory experiments; using established flow cytometry, transcriptomics, and functional assessment methods.

#### Randomization and blinding

Non-applicable. Studies were conducted in a sequence informed by data accrual. Investigators who assessed, measured, or quantified results were commonly blinded to the identities of the experimental groups.

### Cells and antibodies

All cell lines, primary cells, and antibodies are described in the Supplementary Materials and table S8.

### Generation of TCR transductants

TCR α and β chains were amplified by conventional reverse transcription polymerase chain reaction from cDNA derived from PBMCs RNA and cloned into pCSIW (*19*) as a TCRβ–internal ribosomal entry site–TCRα cassette, using the following primers (all sequences read 5′ to 3′): TRBV5-1 (forward): ATGGGCTCCAGGCTGC; TRBV5-4 (forward): ATGGGCCCTGGGCTC; TRBV6-1 (forward): ATGAGCATCGGGCTCCTG; TRBV6-5 (forward): ATGAGCATCGGCCTCCTG; TRBV6-6 (forward): ATGAGCATCAGCCTCCTGTG; TRBV12-3 (forward): ATGGACTCCTGGACCTTCTGC; TRBV12-5 (forward): ATGGCCACCAGGCTCC; TRBV20-1 (forward): ATGCTGCTGCTTCTGCTGC; TRBV29-1 (forward): ATGCTGAGTCTTCTGCTCCTTCTC; TRBC1 (reverse): CTCCACTTCCAGGGCTGC; TRAV24 (forward): ATGGATGGAGAAGAATCCTTTGGCAGC; TRAC (reverse): TCAGCTGGACCACAGCC.

Mutations in the V domain of TCRβ chains were performed by overlap-extension PCR. Lentiviral particles were produced in 293 T cells to transduce J76 cells as described previously (*19*).

### Surface plasmon resonance

TCR α and β chains were fused to an Armenian hamster Fc to generate a heterodimeric Fc-fusion TCR, cloned into the mammalian expression vector pcDNA3.4 (Thermo Fisher Scientific), and transiently cotransfected in CHO cells using the ExpiCHO expression system (Thermo Fisher Scientific). Following harvest by centrifugation, the clarified cell culture supernatant was loaded on a MabSelect SuRe column (Cytiva) and eluted with pH 3.0 elution buffer. The protein A eluate was neutralized, concentrated, and applied to a HiLoad Superdex 200 10/300 GL Size exclusion chromatography column (Cytiva) equilibrated in Dulbecco’s phosphate-buffered saline (PBS) (pH 7.4) for further polishing. Pure fractions containing the peak of interest were pooled.

Interactions between the TCR and α-TRBV6-5 antibodies were analyzed on a Biacore T200 instrument (Cytiva). Antibodies (α-TRBV6-5^PAR^ or α-TRBV6-5^AM^) were immobilized on Series S CM5 chips via α-human Fc antibody (Cytiva). TCR concentration was adjusted to 1000, 500, 250, 125, and 62.5 nM in 1× HBS-EP^+^ running buffer (Cytiva). Single cycle kinetics was run with an association time of 120 s and a final dissociation time of 600 s at 30 μl/min. Sensorgrams were corrected by reference subtraction using the reference flow cell not treated with antibody. BIAevaluation software (Cytiva) was used for data analysis, and kinetics were determined using a 1:1 Langmuir binding model for calculation of the *K*_D_ value. Where faster off rates were observed, the data were fit using a steady-state model.

### Crystal structure

#### Aglycosylated TCR generation

All N-linked glycosylation sites (NXS/T) in the 9B2 TCR [TRAV12-3/TRBV6-5, Protein Data Bank (PDB) 4WWK] were mutated to serine or alanine. DNA encoding the Fc fusions (containing knob and hole mutations for heterodimerization) of α and β chain mutants were synthesized, cloned, and expressed as above. Following harvest by centrifugation, the clarified cell culture supernatant containing the aglycosylated Fc-fused TCR was captured on a MabSelect SuRe column (Cytiva) and eluted with pH 3.0 elution buffer. The protein A eluate was neutralized using 1 M tris-HCl, diluted in cation exchange (CEX) running buffer and loaded on a Mono S 10/100 GL CEX column (Cytiva). The Fc-fused TCR was eluted using a linear salt gradient, and fractions containing the peak of interest were pooled. For the final polishing, the CEX pool was concentrated and applied to a HiLoad 16/600 Superdex 200 pg size exclusion chromatography column (Cytiva) equilibrated in Dulbecco’s PBS (pH 7.4). Pure fractions containing the peak of interest were pooled. The Fc-fused TCR was incubated with FabRICATOR (Genovis Inc.) enzyme immobilized resin followed by collection of fragments via centrifugation. The heterodimeric TCR devoid of Fc was then isolated using CaptureSelect Fc column that captured the Fc while allowing TCR to flow through. The resulting TCR was further used in complexation experiments.

#### Preparation of TCR and Fab complexes

The purified Fab and digested aglycosylated TCR were mixed in a 1.5:1 ratio and incubated overnight at 4°C. The complex was applied to a HiLoad 16/600 Superdex 200 pg size exclusion chromatography column (Cytiva) equilibrated in Dulbecco’s PBS (pH 7.4) to separate complexes from free Fab and TCR. Fractions containing the TCR-Fab complex were pooled and buffer-exchanged into Hepes-buffered saline. The complex was further concentrated to 10 mg/ml for use in crystallization trials.

#### Crystallization of the TCR-Fab complex

Crystallization of the complex was carried out using the sitting drop method on a NT8 robot at 18°C. Initial crystallization trials were carried out using commercial sparse-matrix screens by mixing 100 nl of protein solution with 100 nl of precipitant solution. Six different promising conditions were identified from the initial screens. Optimization screens were set up around the identified conditions by varying the pH, the concentration of precipitants, and the concentrations of salt. A crystalline precipitate was observed in 20% PEG3350 and 0.2 M magnesium sulfate. These conditions for crystallization were further optimized using the additive screen (Hampton Research). Strontium chloride (10 mM) as an additive resulted in well-formed crystals after 5 to 7days. Crystals grew to about 300 to 400 mm in the longest dimension. Crystals were transferred to various solutions for cryoprotection before being frozen in liquid nitrogen. The final crystal that provided the data used to obtain the structure of the complex was cryoprotected in a solution containing 20% PEG3350, 0.2 M magnesium sulfate, and 10% glycerol.

#### Data collection and processing

The diffraction data at a resolution of 2.54 Å were collected at cryogenic temperature (100 K) at the 23-ID-D GM/CA beamline at the Advanced Photon Source (Chicago) with 0.2° oscillation/image. Data reduction was carried out in Xia2 (CCP4) using the DIALS pipeline. Molecular replacement was carried out with Phaser (CCP4) using the structure of the T cell receptor (chains A and B from PDB 4WWK) and the Fab chains (chains H and L from PDB 3PP4) as a search model. A single solution was obtained with a log-likelihood gain (LLG) score of 7292 and a translation function Z (TFZ) score of 38.4. Initially, 50 cycles of rigid body refinement was carried out using REFMAC5 (CCP4). Subsequently, 50 cycles of restrained refinement was carried out using REFMAC5 (CCP4). Several cycles of manual rebuilding in COOT and restrained refinement using REFMAC5 (CCP4) were carried out. Water molecules were added to the model, and a final round of restrained and TLS refinement was carried out using REFMAC5. The final *R*_work_ and *R*_free_ values were 0.278 and 0.325, respectively. For electron density mapping, the 2mFo-DFc composite omit map (*66*) was contoured to 2 root mean square deviation and generated using phenix.refine in PHENIX.

### Flow cytometry

For analysis of surface markers expression, cells were resuspended in PBS and stained with Zombie NIR (1:1000 dilution; BioLegend) at room temperature for 15 min and then washed in fluorescence-activated cell sorting (FACS) buffer [PBS supplemented with 10% fetal calf serum and 2 mM EDTA (EMD Millipore)]. Cells were stained with the indicated antibodies for 30 min at 4°C, washed twice, and resuspended in FACS buffer for analysis.

For intracellular cytokines and GZMB staining, cells were pretreated with Monensin (BioLegend) at 1:1000 dilution, 24 hours before cells were collected and stained for extracellular markers (above). Cells were fixed (CellFix, BD), washed twice with intracellular staining permeabilization wash buffer (BioLegend), and stained with antibodies against IFN-γ, TNFα, GM-CSF, and GZMB (BioLegend). Cells were further washed twice with intracellular staining permeabilization wash buffer (BioLegend) before resuspension in FACS wash buffer for analysis.

For T-BET and FOXP3 staining, cells were fixed with the Foxp3/transcription factor staining buffer set (eBioscience) and washed twice in permeabilization buffer (eBioscience) before staining with Allophycocyanin (APC) anti–T-BET and phycoerythrin (PE) anti-human FOXP3 antibodies (BioLegend).

For IRF4 staining, cells were fixed with the True-Nuclear Transcription Factor Buffer Set (BioLegend) and washed twice with permeabilization buffer (BioLegend) before staining with PE anti-IRF4 antibody (BioLegend).

For cell cycle analysis, cells were stained after permeabilization with Hoescht 33342, as described previously (*67*). Samples were acquired on a BD LSR Fortessa flow cytometer, and results were analyzed using FlowJo v9 and v10 (BD).

### TCR sequencing

Total RNA was extracted from purified T cells that were either unstimulated (baseline) or stimulated [day 7 culture and supplemented with recombinant human interleukin 2 (hIL-2) up to day 11] with 10 nM plate-bound α-CD3ɛ^OKT3^, α-TRBV6-5^PAR^, or α-TRBV6-5, using the Maxwell SimplyRNA Kit (Promega). RNA was quantified using the Qubit High Sensitivity RNA Assay (Thermo Fisher Scientific) and quality-checked using Agilent Tapestation. Sequencing libraries were generated using the SMARTer Human TCR α/β Profiling Kit (Takara) according to the manufacturer’s protocol. Final libraries were then pooled and sequenced (paired end, 300 bp) on Illumina MiSeq. Data generated were demultiplexed, and FastQC was performed after trimming. MiXCR pipeline tool was used to specifically align sequence reads to TCR germline segments of TRA, TRB, and CDR3 sequences. TRBV and TRBJ sequences are counted and represented as frequencies.

### Cytokine production analysis

The LegendPlex Human T_H_ (12-plex) and Human Anti-Virus Response (13-plex) panels (BioLegend) were used following the instructions provided by the manufacturer with minor modifications. Supernatants from T cell stimulation assays (day 7), mixed beads, detection antibodies, and streptavidin-PE were diluted twofold using assay buffer before the assay, which was performed in V-bottom 96-well plates. Twenty-five microliters of each diluted reagent was used for the following steps. Samples were acquired on a BD LSR Fortessa X20. Data analysis was performed using the LegendPlex data analysis software v.8 (BioLegend). Final quantities (nanograms) of each cytokine were derived from the concentrations provided by the software analysis, adjusted to account for dilutions and the total volume of culture (200 μl).

### Single-cell RNA sequencing

Control (Unstim) cells were thawed 24 hours before processing and rested in complete media (above) without cytokines. Stimulated cells were cultured in antibody-coated plates as described above for 7 days. All cells were then harvested and resuspended in ice-cold PBS supplemented with 1% bovine serum albumin (BSA) and 2 mM EDTA (staining buffer) and processed using a dead cell removal kit according to the manufacturer’s instructions (Miltenyi). Flow-through was supplemented with Fc block (1:10 dilution; BioLegend), and then cells from each condition were pooled and labeled with a cocktail of barcoded antibodies against various markers and for cell hashing for 30 min at 4°C, washed three times with staining buffer, and resuspended in 50 μl of ice-cold PBS supplemented with 0.04% BSA at ≤10^6^ cells/ml for loading. Cells were processed for gene expression and immune profiling using the 10X Genomics Chromium Single Cell platform. Single-cell libraries were generated using the Chromium Single-Cell 5′ Reagent Kits v2 assay following the manufacturer’s instructions, quantified, pooled, and sequenced on the NextSeq 2000 platform.

Raw reads were mapped with CellRanger (v.6.0.0) and imported into Seurat for downstream analyses. For quality control, cells with unique molecular identifiers (UMI) counts between 300 and 4000 and with less than 5% reads mapping to mitochondrial genes were retained. A total of 15,597 cells were used for downstream analyses. Protein abundance data (antibody-derived tag) were normalized using a centered log-ratio approach prior scaling. Normalized and scaled data were for computing principal components analysis and uniform manifold approximation and projection (UMAP) coordinates and performing clustering analyses using Seurat’s Louvain’s algorithm at a resolution of 0.25. Information about TRBV usage was extracted from VDJ data, so that cells using TRBV6-1, TRBV6-5, TRBV6-2, and TRBV10-3 were used to group cells targeted by α-TRBV6-5^AM^ and vice versa. Differential gene expression was performed using Seurat’s default Wilcox test with a minimum of three cells per group, excluding genes expressed in less than 10% of the cells. Adjusted *P* values reported by Seurat use Bonferroni correction at a significance threshold of *P* < 0.05 and were used to determine differentially expressed genes (DEG) between the two conditions. Where imputed data are used, raw counts were imputed using the Rmagic package (v2.0.3, https://cran.r-project.org/web/packages/Rmagic/index.html). The list of transcription factors used in this manuscript was downloaded from the gene set enrichment analysis (GSEA) molecular signatures database (MsigDB) (http://gsea-msigdb.org). The significance of the overlap between the transcription factor groups was calculated using hypergeometric distribution (R’s phyper function) with the universe represented by all transcription factors (*n* = 1536) and testing the null hypothesis that there is no significant enrichment of the genes in the overlap between the two groups.

### NanoString

T cells from day 7 stimulation assays were harvested and resuspended in cold FACS buffer and stained for cell sorting (BD Aria II) of four subsets per sample directly into lysis buffer for RNA extraction (RNeasy Mini kit, Qiagen), based on the expression of CD4, CD8, TRBV6-5, and TRBV “control” pool consisting of a mix of cells positive for staining with α-TRBV5-1 (Vβ5.1, Miltenyi), α-TRBV12-3/4 (Vβ8, Beckman Coulter), α-TRBV19 (Vβ17, Beckman Coulter), and α-TRBV20-1 (Vβ2, Beckman Coulter).

RNA concentrations were measured using NanoDrop, adjusted to 15 ng/μl, and processed for analysis on a Flex Gen 2 nCounter (NanoString) following the manufacturer’s instructions. Probes for expression analysis were from the Human chimeric antigen receptor T cell (CAR-T) Characterization Panel supplemented with probes (Panel Plus, NanoString) for *CD101*, *FCER1G*, *HOPX*, *ID3*, *ITGAE*, *KLF2*, *LGALS1*, *ZBED2*, *ZBTB7A*, and *ZNF683*. Data were analyzed using nSolver v4.0 (NanoString) following the manufacturer’s instructions.

## Supplementary Materials

### This PDF file includes:

Supplementary Methods Fig. S1 to S8 Legends for tables S1 to S8 References
