# Supplementary material for: Innate TCRβ-chain engagement drives human T cells toward distinct memory-like effector phenotypes with immunotherapeutic potentials
Source: Sci Adv. 2023 Dec 6;9(49):eadj6174. doi: 10.1126/sciadv.adj6174 (PMC10699787; doi:10.1126/sciadv.adj6174)
Supplement: Supplementary file 1 — Supplementary Methods Fig. S1 to S8 Legends for tables S1 to S8 References [file sciadv.adj6174_sm.pdf]

Science Advances
AAAS

Supplementary Materials for

Innate TCRβ-chain engagement drives human T cells toward distinct memory-like effector phenotypes with immunotherapeutic potentials

Pierre Vantourout *et al.*

Corresponding author: Pierre Vantourout, pierre.vantourout@kcl.ac.uk;
Adrian C. Hayday, adrian.hayday@kcl.ac.uk

Sci. Adv. **9**, eadj6174 (2023)
DOI: 10.1126/sciadv.adj6174

This PDF file includes:

Supplementary Methods
Figs. S1 to S8
Legends for tables S1 to S8
References

Other Supplementary Material for this manuscript includes the following:

Tables S1 to S8

Supplementary Materials for: Innate TCRβ-chain Engagement Drives Human T cells Toward Distinct Memory-like Effector Phenotypes with Immunotherapeutic Potentials

Pierre Vantourout[1,2,*], Josephine Eum[1,2], María Conde Poole[1,2], Thomas S. Hayday[1,2], Adam G. Laing[1,2], Khiyam Hussain[1,2], Rosamond Nuamah[3], Shichina Kannambath[3], Jacques Moisan[4], Allart Stoop[5], Sebastiano Battaglia[6], Roya Servattalab[4], Jonathan Hsu[4], Andrew Bayliffe[4], Madan Katragadda[4], Adrian C. Hayday[1,2,*].

Supplementary Methods:

Cell lines and primary cells.

HEK293T subclone 17 (hereafter referred to as 293T) and J76 cells were from the ATCC. 293T cells were maintained in maintained in DMEM supplemented with 4.5 g/l D-glucose, L-glutamine, 10% heat-inactivated fetal calf serum (HI-FCS), penicillin (50 units/ml), and streptomycin (50 μg/ml) (P/S). J76 cells were maintained in RPMI 1640 supplemented with L-glutamine, 10% HI-FCS and 1% P/S.

PBMC were isolated from cone blood samples provided by the NHS Blood and Transplant service (London, UK) by Ficoll gradient centrifugation. T cells were enriched using Pan T cell isolation kits (Miltenyi). In all stimulation experiments, PBMC or T cells were maintained in X-VIVO 15 supplemented with 5% heat-inactivated human AB serum, 1 mM sodium pyruvate in the presence of IL-2 (100 U/ml) and IL-15 (10 ng/ml) for 7 days, unless specified otherwise. In experiments where cell proliferation was monitored, T cells were labelled with Cell Trace Violet (CTV, 1 μg/ml) prior to stimulation.

All cell culture reagents were from Thermo Fisher, except FCS, human AB serum, Toxic shock Syndrome Toxin-1 (TSST-1) (Merck); recombinant human IL-2 (Novartis Pharmaceuticals, via Guy's Hospital); and recombinant human IL-15 (Biolegend).

Antibodies

Commercial antibodies used in this study for cell stimulation, flow cytometry and CITE-Seq are listed in Supplementary Table 8. Humanized antibodies against TRBV5-1, TRBV6-5, TRBV12-3/4 and TRBV20-1 were derived from available mouse monoclonal antibodies (REA1062, Miltenyi; H131, Biolegend; I6G8, Thermo Fisher; and MPB2D5, Beckman Coulter; respectively) and defined as Parental (PAR).

The TRBV6-5 antibody was optimized for affinity, cross-reactivity to non-human primates and stability using molecular evolution strategies. Three rounds of molecular evolution using five different phage/yeast libraries were employed. Each round constituted creating a DNA library of scFv mutants, expressing the library on the surface of M13 filamentous phage and/or yeast (Saccharomyces cerevisiae), enriching the phage or yeast population for improved clones by incubating the library at different conditions (low antigen concentration, competition for scarce antigen in the presence of excess competitor, high-temperature stress) nonpermissive for the parental clone followed by high-throughput individual clone screening by ELISA and/or flow cytometry. For the thermostability branch, prior to incubation with 10 nM antigen, library phage was incubated at temperatures at or above the Tm. For affinity screens, bacterial periplasmic extracts containing soluble scFv mutants were tested by ELISA against coated huTRBV6-5 and cynomolgus TRBV antigen in the absence (direct ELISA) and presence of excess parental antibody (competitive ELISA). The final clones derived from the screens were cloned into an expression vector that allowed generation of monovalent scFv-Fc fusion. Following purification, antibodies were

tested for binding to human and cynomolgus antigen by Surface plasmon resonance and thermostability by Differential scanning fluorimetry. The variant that showed the best human/cynomolgus cross reactivity and thermal stability (defined as affinity matured, AM) was chosen as lead for further studies.

For cell stimulation, tissue culture plates were coated with antibodies (10 µg/ml unless specified otherwise) in PBS for 1 h at 37˚C, then washed twice with PBS.

Supplementary Figures:

Supplementary Figure 1

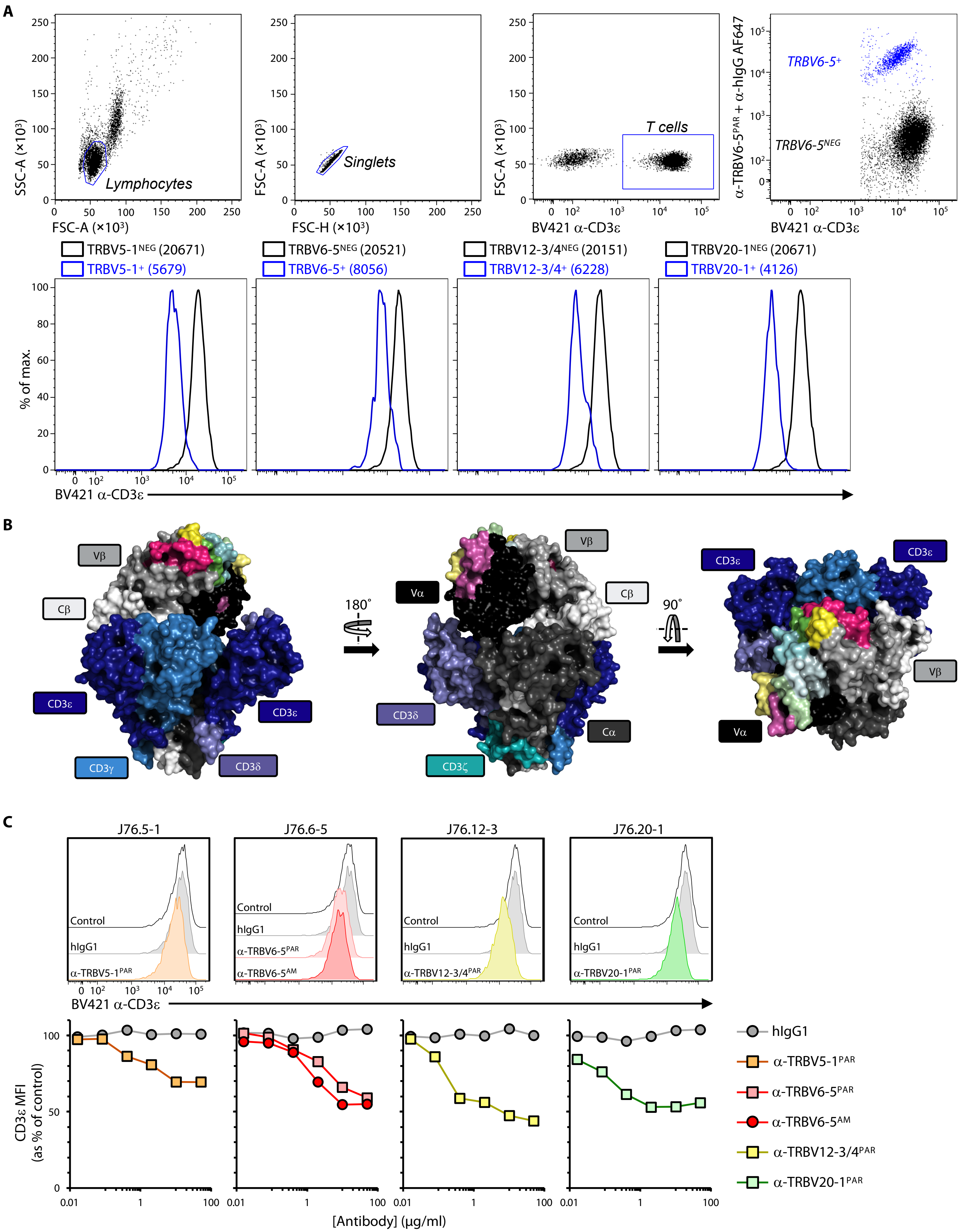

Supplementary Figure 1 (continued)

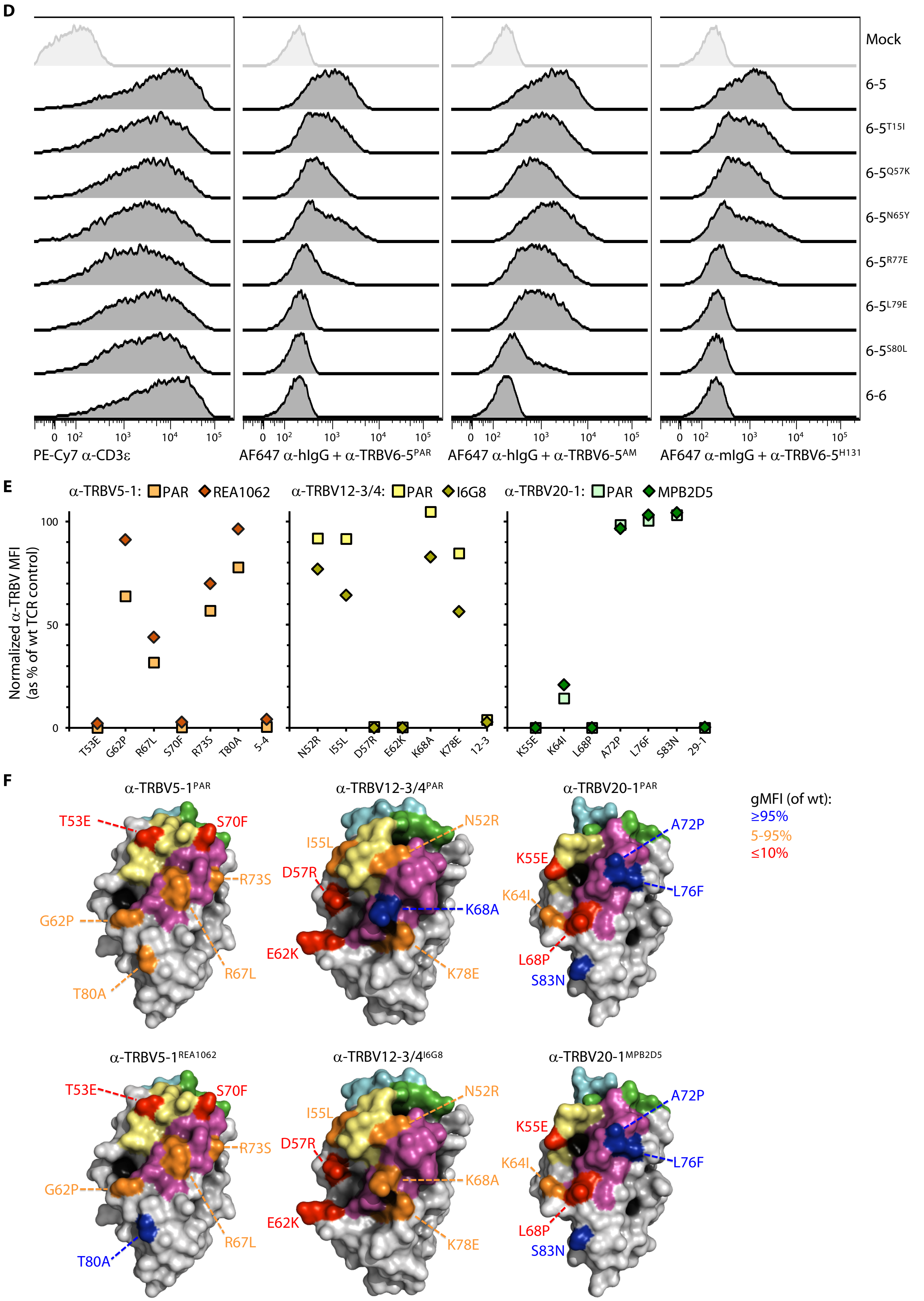

Supplementary Figure 1. Analysis of the binding of α-TRBV antibodies.

A. Flow cytometry analysis of the interference between α-TRBV and α-CD3ε antibodies. Dot plots (top row) show the gating strategy. Overlayed histograms (bottom row) show the consistently lower α-CD3ε staining of cells co-stained by each α-TRBV antibodies (blue) compared to other subsets (black). Numbers indicate α-CD3ε MFI. Representative of 15 donors. **B.** Full TCR complex structure showing that the accessible region of the Vβ domain (grey) is located on the same side as and on top of the CD3ε subunits (dark blue), suggesting that the binding of α-TRBV antibodies interferes with α-CD3ε staining. Derived from PDB 6JXR (*69*). CDR1, CDR2, CDR3 and HV4 regions are highlighted in green, yellow, cyan and magenta, respectively. **C.** J76 cells expressing the indicated TCR were stained for CD3 expression without (Control) or with prior incubation with increasing doses of the indicated antibodies. Primary flow cytometry histograms for the maximum dose (100 μg/ml) and summary data are shown on the top and bottom rows, respectively. Representative of two independent experiments. **D.** Representative histograms showing the staining of the indicated TRBV6-5 variants and TRBV6-6 negative control by the indicated antibodies. Related to Fig 1C. **E.** Summary flow cytometry analysis of the staining of the indicated TRBV5-1, TRBV12-3 and TRBV20-1 mutants and their respective negative controls (TRBV5-4, TRBV12-5 and TRBV29-1). MFI were normalized to that of α-CD3ε and expressed as % of the wild-type (wt) control stainings. Representative of 3 independent transductions. **F.** Mapping of the aminoacids mutated in D. The Vβ domain structures were derived from PDB 5BRZ (TRBV5-1) (*70*), 6UK4 (TRBV12-3) (*71*), and 4PJ8 (TRBV20-1) (*72*). The CDR1, CDR2, CDR3 and HV4 regions are highlighted in green, yellow, cyan and magenta, respectively.

Supplementary Figure 2

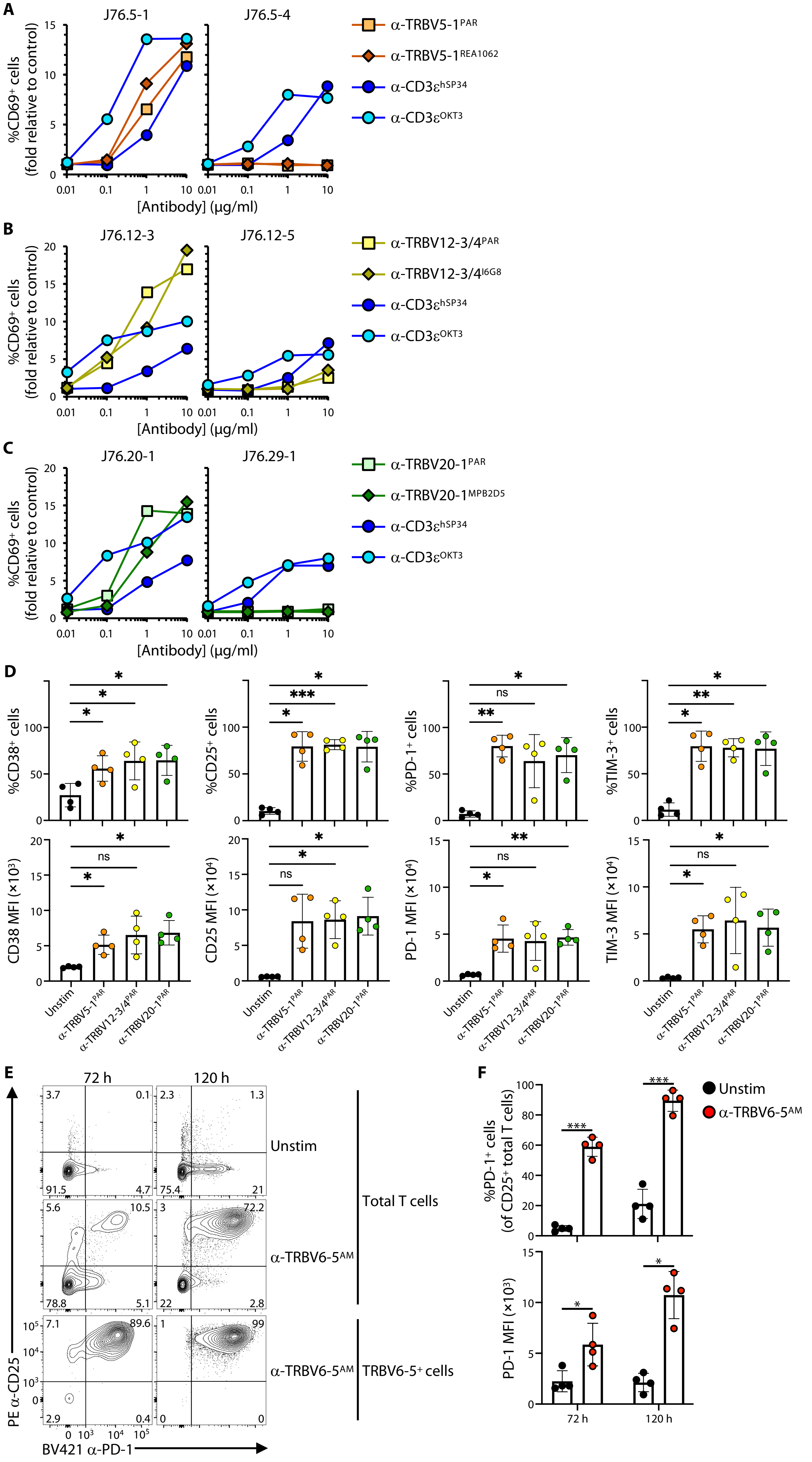

Supplementary Figure 2. Functional assessment of α-TRBV antibodies.

A-C. J76 cells co-expressing a TRAV24$^+$ TCRα chain and a (A) TRBV5-1$^+$ (J76.6-5) or TRBV5-4$^+$ (J76.5-4), (B) TRBV12-3$^+$ (J76.12-3) or TRBV12-5$^+$ (J76.12-5), (C) TRBV20-1$^+$ (J76.20-1) or TRBV29-1$^+$ (J76.29-1) TCRβ chain we incubated for 4 h at 37˚C with increasing concentrations of the indicated plate-bound antibodies, then stained for analysis of CD69 upregulation by flow cytometry. Representative of 2 independent experiments. **D.** Purified T cells were cultured as in Fig 2C,D without (Unstim) or with the indicated α-TRBV antibodies then stained for the indicated activation markers for analysis by flow cytometry. Percentages of positive cells and MFI were determined after gating on total T cells (Unstim) or the subsets corresponding to the α-TRBV antibodies. Data are shown as mean +/- SD. ns, not significant; *, p<0.05; **, p<0.01; ***, p<0.001 (paired ANOVA with Dunnett's method, comparing α-TRBV6-5^{AM} to every other condition individually). **E.** Purified T cells were stained for CD25 and PD-1 after culture as in Fig 2C,D without (Unstim, top row) or with plate-bound α-TRBV6-5^{AM} (middle and bottom rows) at the indicated time points for analysis by flow cytometry. The bottom row shows CD25 and PD-1 expression in α-TRBV6-5^{AM}-stimulated cells at the indicated time points, after gating on TRBV6-5$^+$ cells. Representative of n=4 donors. **F.** Summary data of PD-1 expression (% positive cells, top; MFI, bottom) by CD25$^+$ total T cells for all 4 donors, shown as mean +/- SD. ns, not significant; *, p<0.05; p<0.01; ***, p<0.001 (paired ANOVA with Dunnett's method).

Supplementary Figure 3

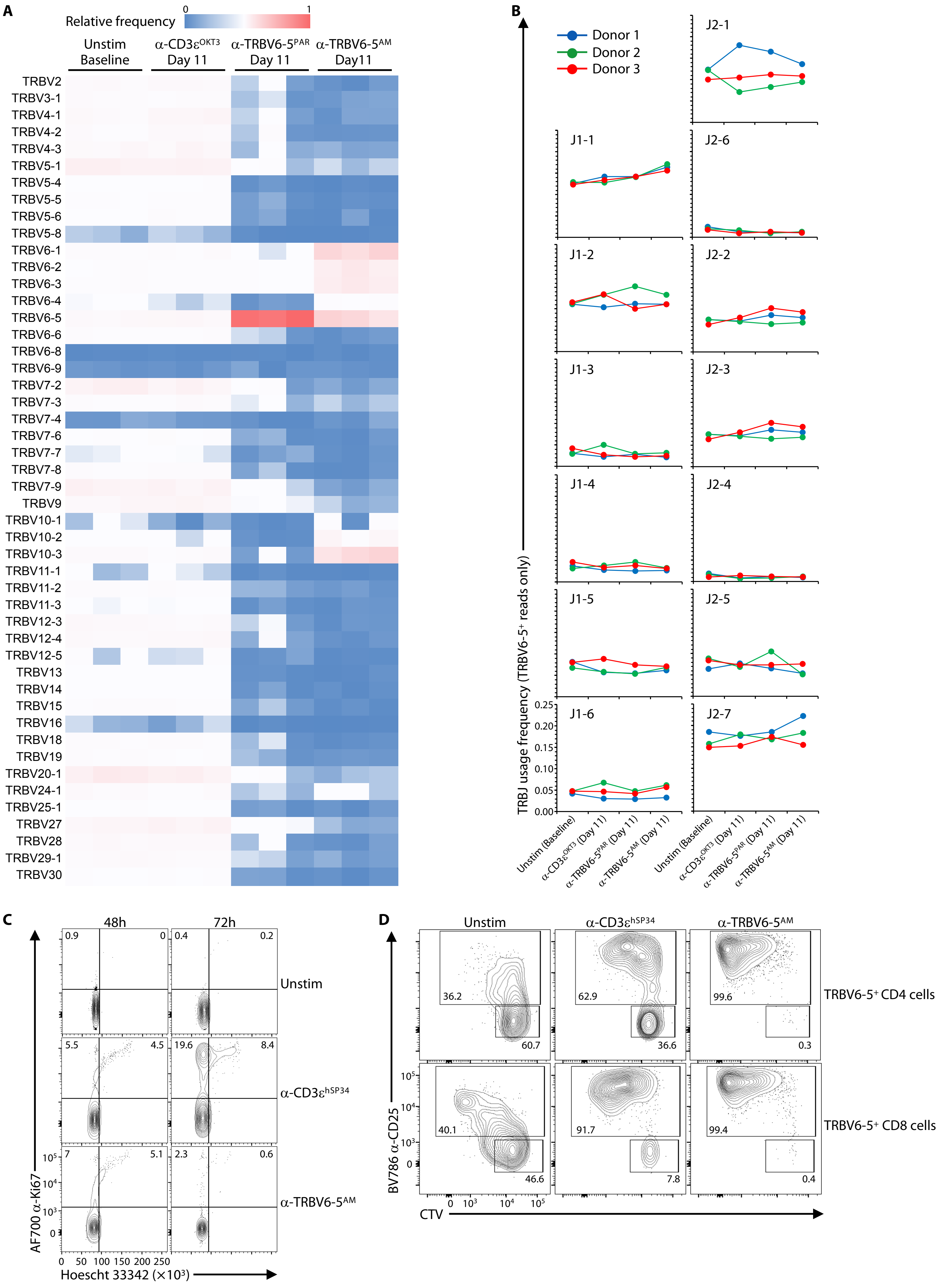

Supplementary Figure 3. Specific expansion of Vβ subsets.

A. Purified T cells from 3 donors were analysed for TRBV usage by TCR sequencing before (Unstim Baseline) or after expansion with the indicated antibodies for 7 days and supplemented with IL-2 for another 4 days (11 days total) to evaluate the specific TRBV subset enrichment driven by α-TRBV6-5^{PAR} and α-TRBV6-5^{AM} antibodies. **B.** Sequencing data from A was further analysed for J usage in TRBV6-5^{+} sequences only, to verify that α-TRBV6-5 antibodies do not drive a clonal expansion compared to α-CD3ε antibodies. **C.** Representative cell cycle analysis contour plots from one donor, related to Fig 3C. **D.** Representative contour plots for one donor (C023) showing proliferation (CTV dilution) in CD25^{+} (activated) CD4 (left) and CD8 (right) cells following a 5-day culture without (Unstim) or with the indicated plate-bound antibodies. Related to Fig 3D.

Supplementary Figure 4

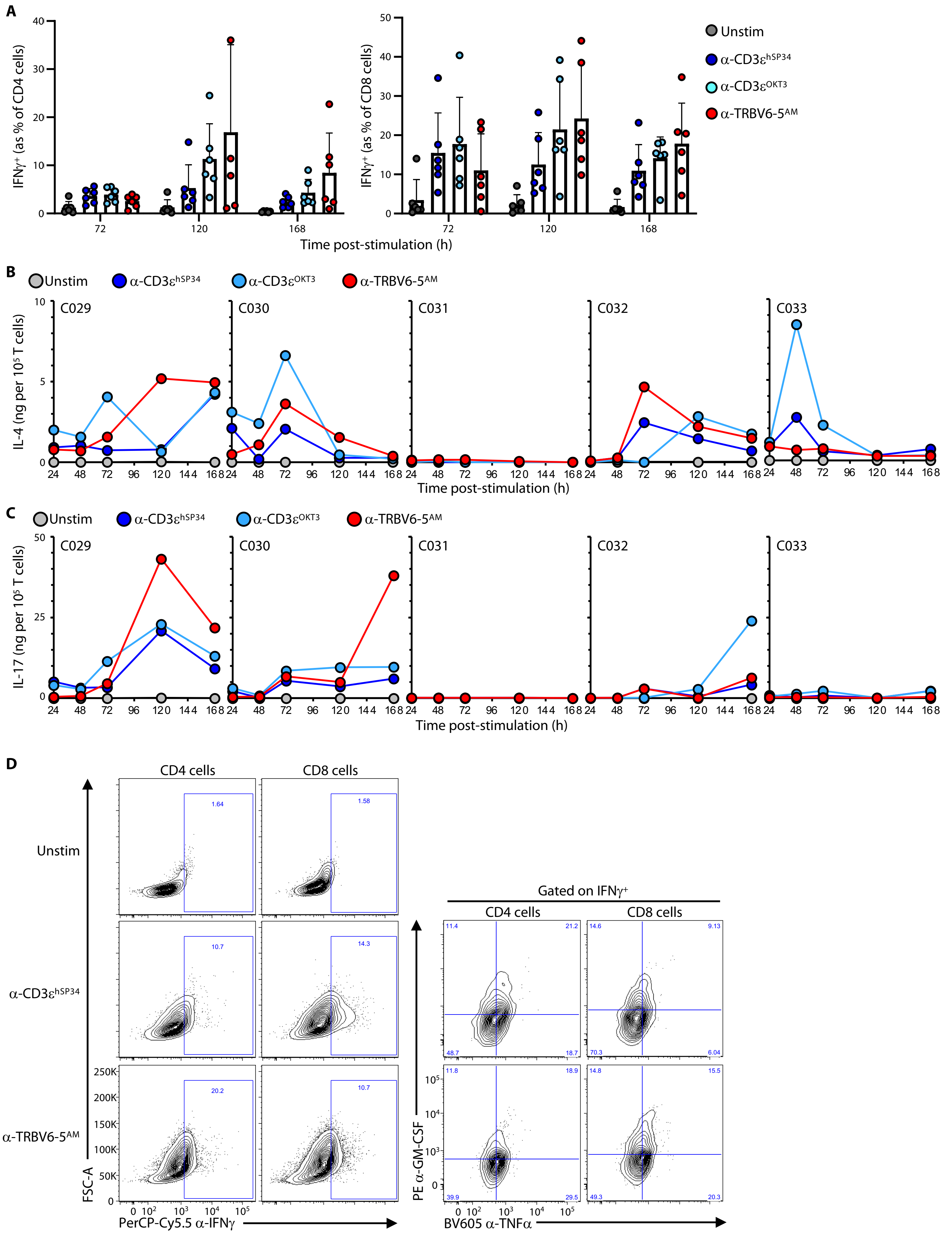

Supplementary Figure 4 (continued)

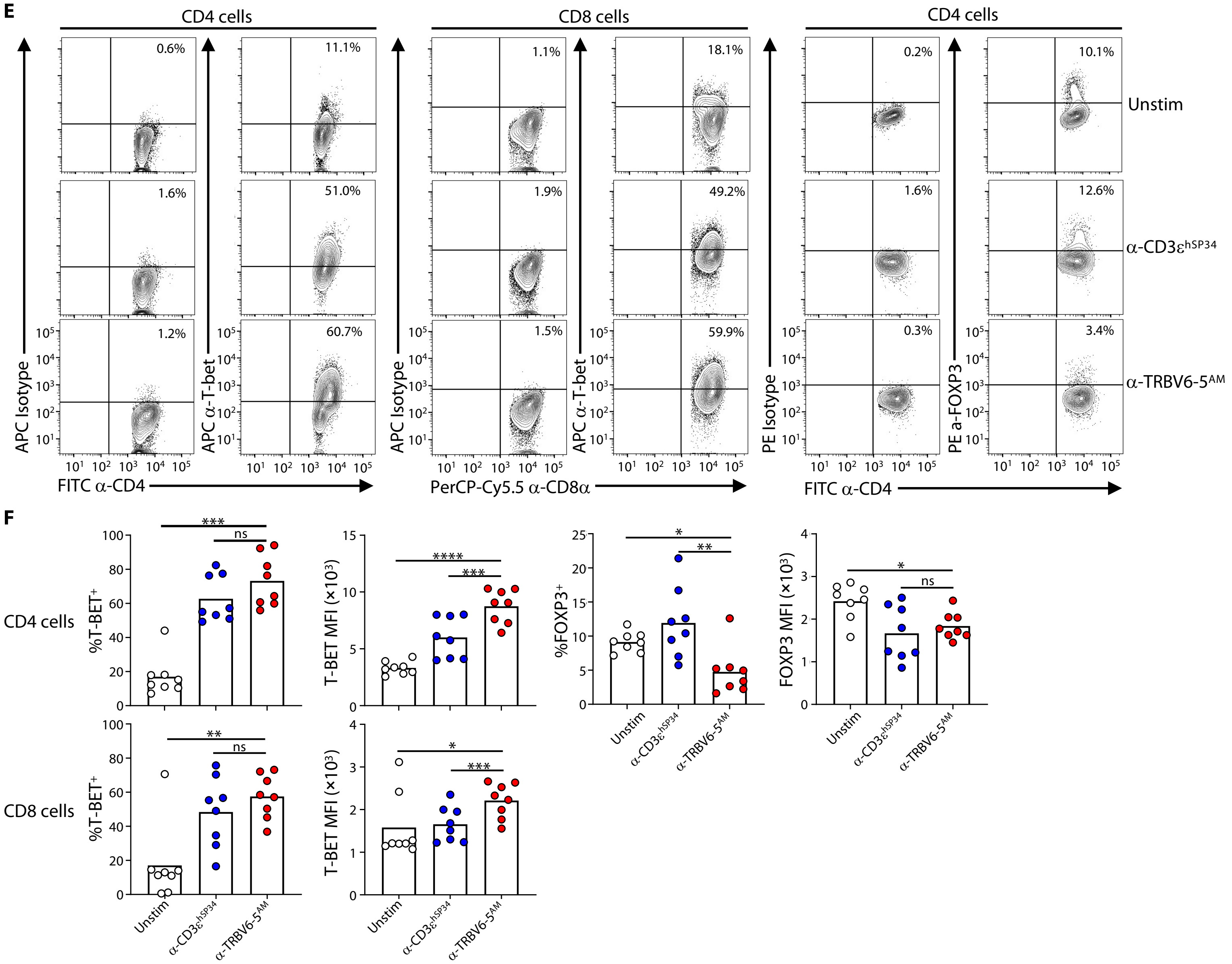

Supplementary Figure 4. Evaluation of effector responses.

A. Summary data of intracellular IFN-γ staining in CD4$^+$ (left) and CD8$^+$ (right) cells at 72, 120, and 168 h post-stimulation. Related to Fig 4B. **B,C.** Purified T cells from n=5 donors were cultured as in Fig 2C,D without (Unstim) or with the indicated plate-bound antibodies and supernatants were collected at the indicated time points to evaluate the cumulative secretion of IL-4 (B) and IL-17 (C) over the indicated time points by flow cytometry using the T_H response and anti-viral response Legendplex assays, related to Fig 4C. The complete Legendplex dataset is provided in Supplementary Table 2. **D.** Representative contour plots for one donor showing GM-CSF and TNFα expression (right columns) after gating on IFNγ$^+$ CD4 and CD8 cells (left columns). Related to Fig 4D. **E,F.** Purified T cells were cultured as in Fig 2C,D without (Unstim) or with the indicated plate-bound antibodies for 7 days then stained intracellularly for T-BET or a matching isotype control for analysis by flow cytometry in CD4 (left) and CD8 (center) cells, and for FOXP3 or a matching isotype control for analysis by flow cytometry in CD4 (right) cells. Representative plots of n=8 donors are shown in E. Summary data in F are shown as mean +/- SD. ns, not significant; *, $p<0.05$; **, $p<0.01$; ***, $p<0.001$; ****, $p<0.0001$ (paired ANOVA with Dunnett's method, α-TRBV6-5^{AM} to every other condition individually).

Supplementary Figure 5

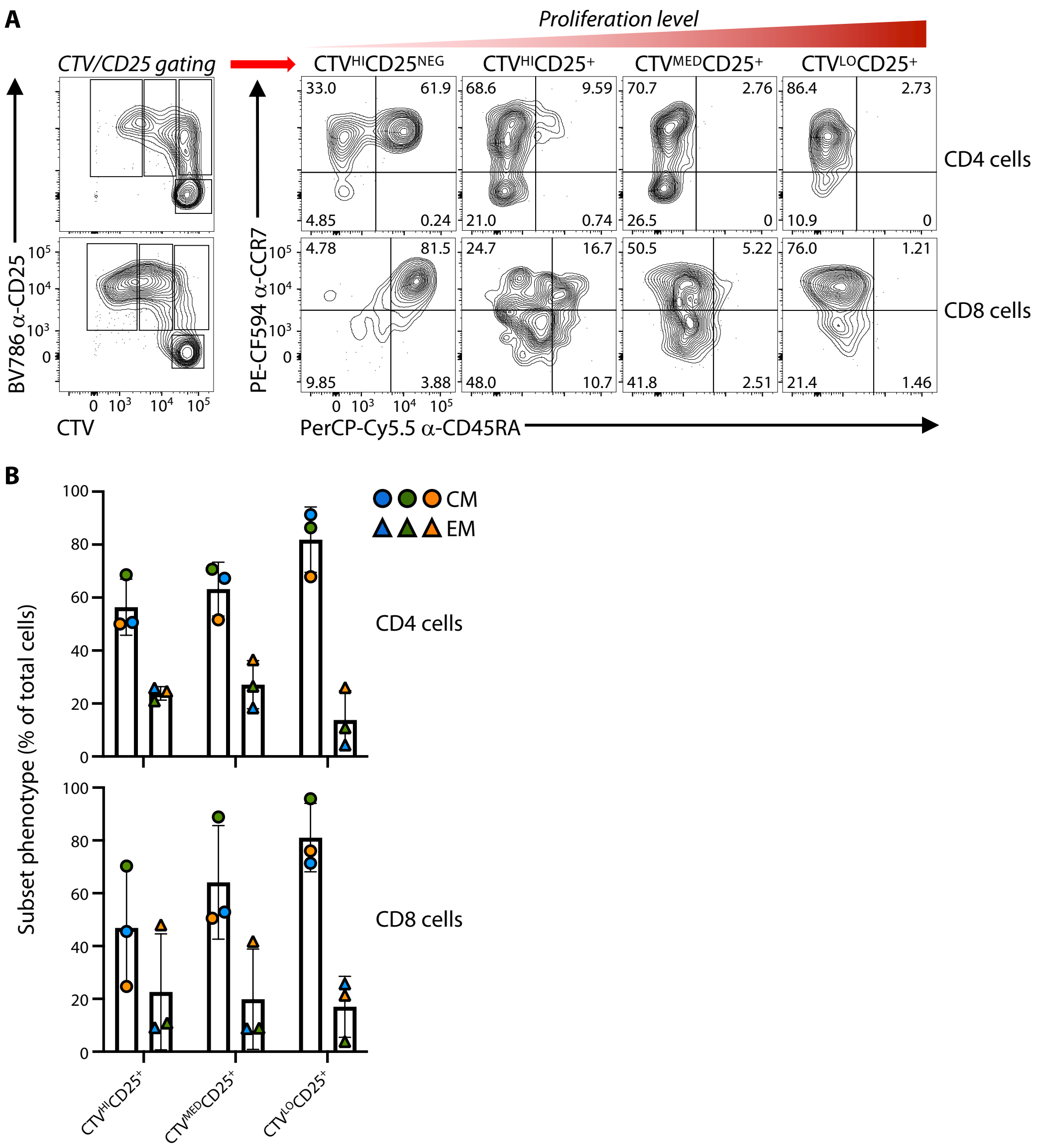

Supplementary Figure 5. "CM-like" phenotype as a function of proliferation.

A. Purified T cells were labelled with CTV then cultured for 5 days with plate-bound α-CD3ε^{hSP34} as in Fig 2C,D, then stained for CD45RA, CCR7, and CD25 to determine the proportion of naïve and memory phenotypes as a function of the level of activation and proliferation by flow cytometry. Representative contour plots for CD45RA vs. CCR7 (right) expression by TRBV6-5^{+} cells after gating (red block arrow) on the CTV^{HI}CD25^{NEG} (non-activated), CTV^{HI}CD25^{+} (low proliferation), CTV^{MED}CD25^{+} (moderate proliferation), and CTV^{LO}CD25^{+} (robust proliferation) subsets (left) for CD4 (top row) and CD8 cells (bottom row) are shown for one donor. **B.** Summary data of the relative percentages of CM and EM cells in the indicated CTV/CD25 gates (CTV^{HI}CD25^{NEG} cells were omitted due to the very low number of CM and/or EM events in some cases) for n=3 donors, identified by colour-coding.

Supplementary Figure 6

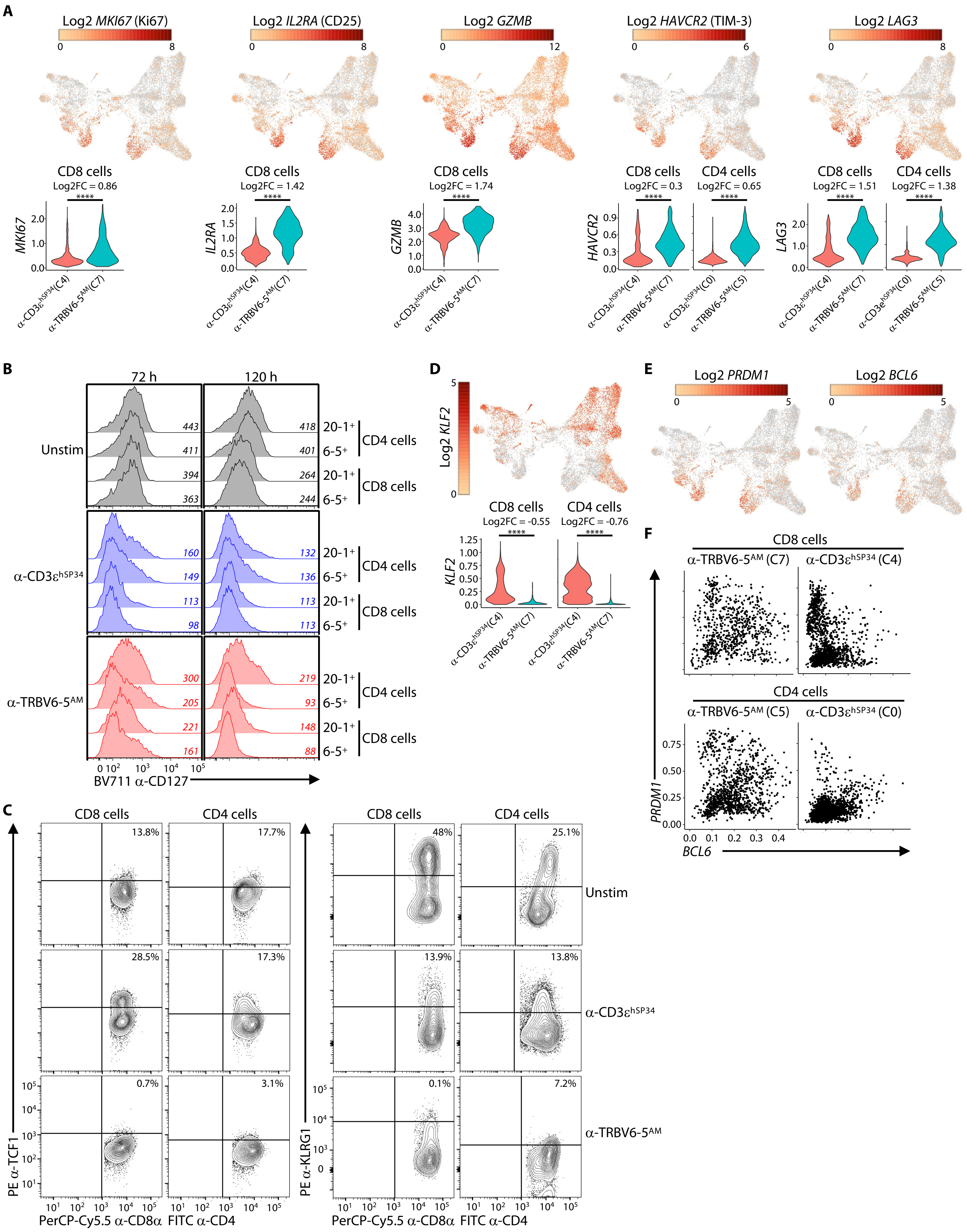

Supplementary Figure 6. Transcriptomics- and flow cytometry-based analysis of α-TRBV-stimulated cells.

A. Expression of the indicated genes visualised in the UMAP (top row) and as violin plots (imputed expression, bottom row). Log2FC, Log2 fold-change; ****, $p<0.0001$ (Wilcoxon rank sum test with Bonferroni correction, from the DEG analysis). Related to Fig 6D. **B.** Representative flow cytometry histograms showing the expression of CD127 at the indicated time points and in the indicated cell subsets from one donor. Numbers in italic indicate MFI. Related to Fig 6E. **C.** Representative flow cytometry contour plots showing the intracellular expression of TCF1 and cell-surface expression of KLRG1 and for one donor. Related to Fig 6G (TCF1) and Fig 6I (KLRG1). **D.** Expression of *KLF2* visualised in UMAP plots (top row) and violin plots (imputed expression, bottom row). Log2FC, Log2 fold-change; ****, $p<0.0001$ (Wilcoxon rank sum test with Bonferroni correction, from the DEG analysis). **E,F.** Expression of *PRDM1* (BLIMP1) and *BCL6* visualised in UMAP plots (E) and their co-expression in scatterplots (imputed expression, F).

Supplementary Figure 7

A

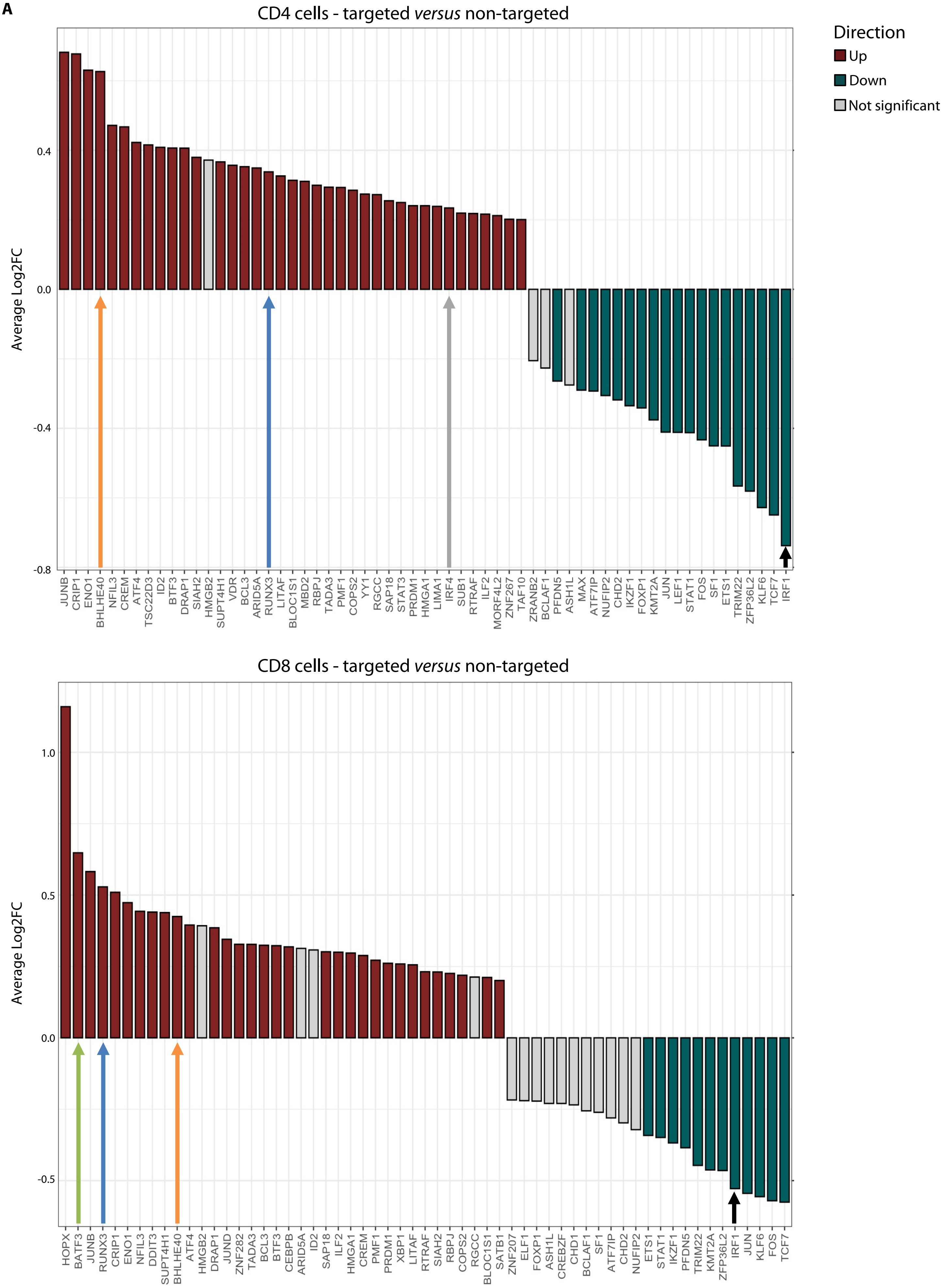

Supplementary Figure 7 (continued)

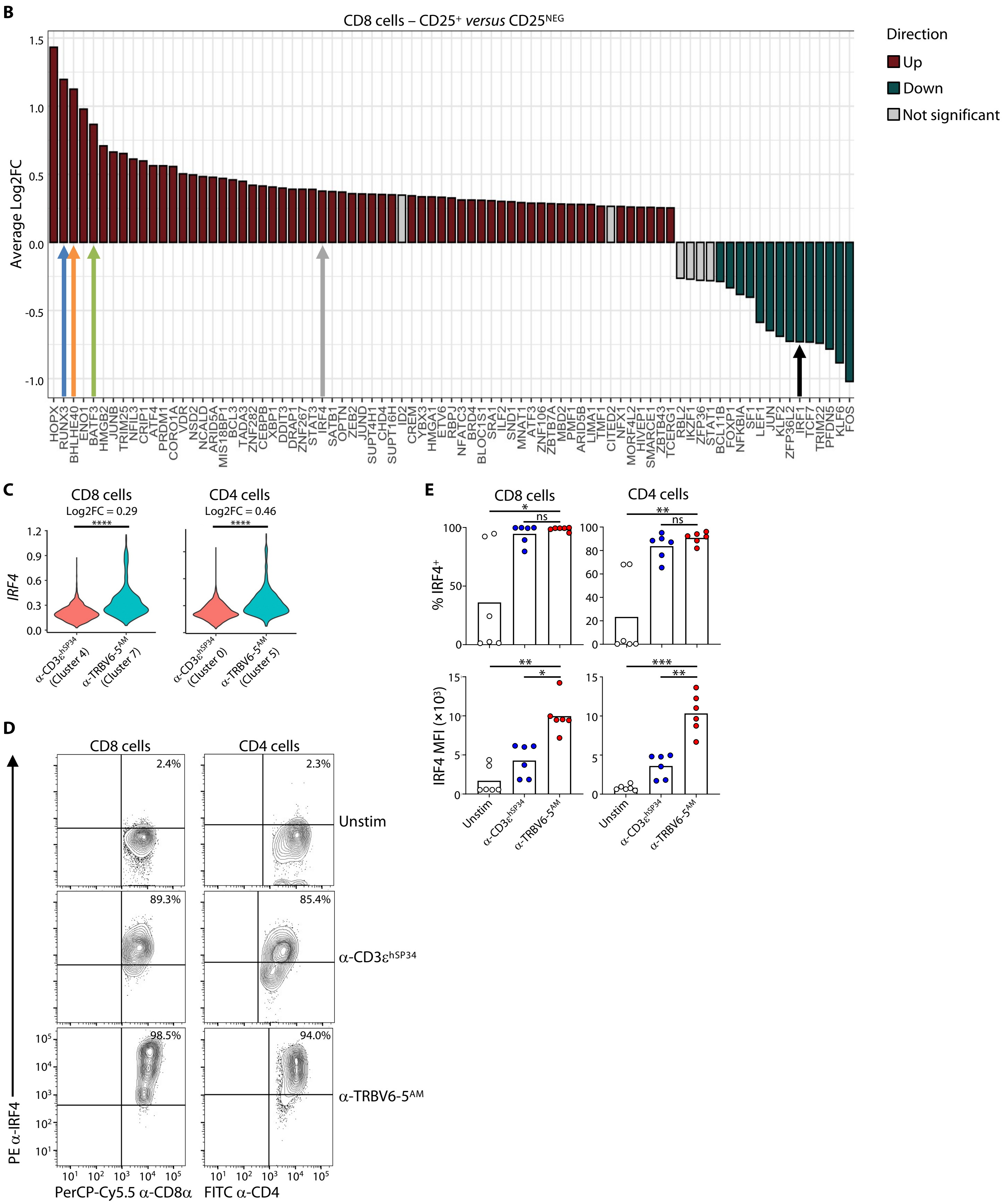

Supplementary Figure 7. Identification of α-TRBV-regulated transcription factors.

A. Waterfall plot showing the Log2FC of transcription factors differentially expressed in α-TRBV6-5^{AM}-targeted *versus* -non-targeted cells in CD4 (top) and CD8 (bottom) cells. Related to Fig 7A. **B.** Waterfall plot showing the Log2FC of transcription factors differentially expressed in α-TRBV6-5^{AM}-stimulated cells, comparing CD25^{+} *versus* CD25^{NEG} CD8 cells. Related to Fig 7A. **C.** Violin plots showing the expression of *IRF4* (imputed expression). Log2FC, Log2 fold-change; ****, p<0.0001 (Wilcoxon rank sum test with Bonferroni correction, from the DEG analysis). **D,E.** Purified T cells were cultured for 7 days without (Unstim) or with the indicated plate-bound antibodies then intracellularly stained for analysis of IRF4 expression by flow cytometry, after gating on CD4 or CD8 cells (right and left columns respectively). Plots representative of n=6 donors are shown in D. Summary data in E are the mean of the percentage of IRF4^{+} (top row) cells, and their MFI (bottom row). ns, not significant; *, p<0.05; **, p<0.01; ***, p<0.001 (paired ANOVA with Dunnett's method, comparing α-TRBV6-5^{AM} to every other condition individually).

Supplementary Figure 8

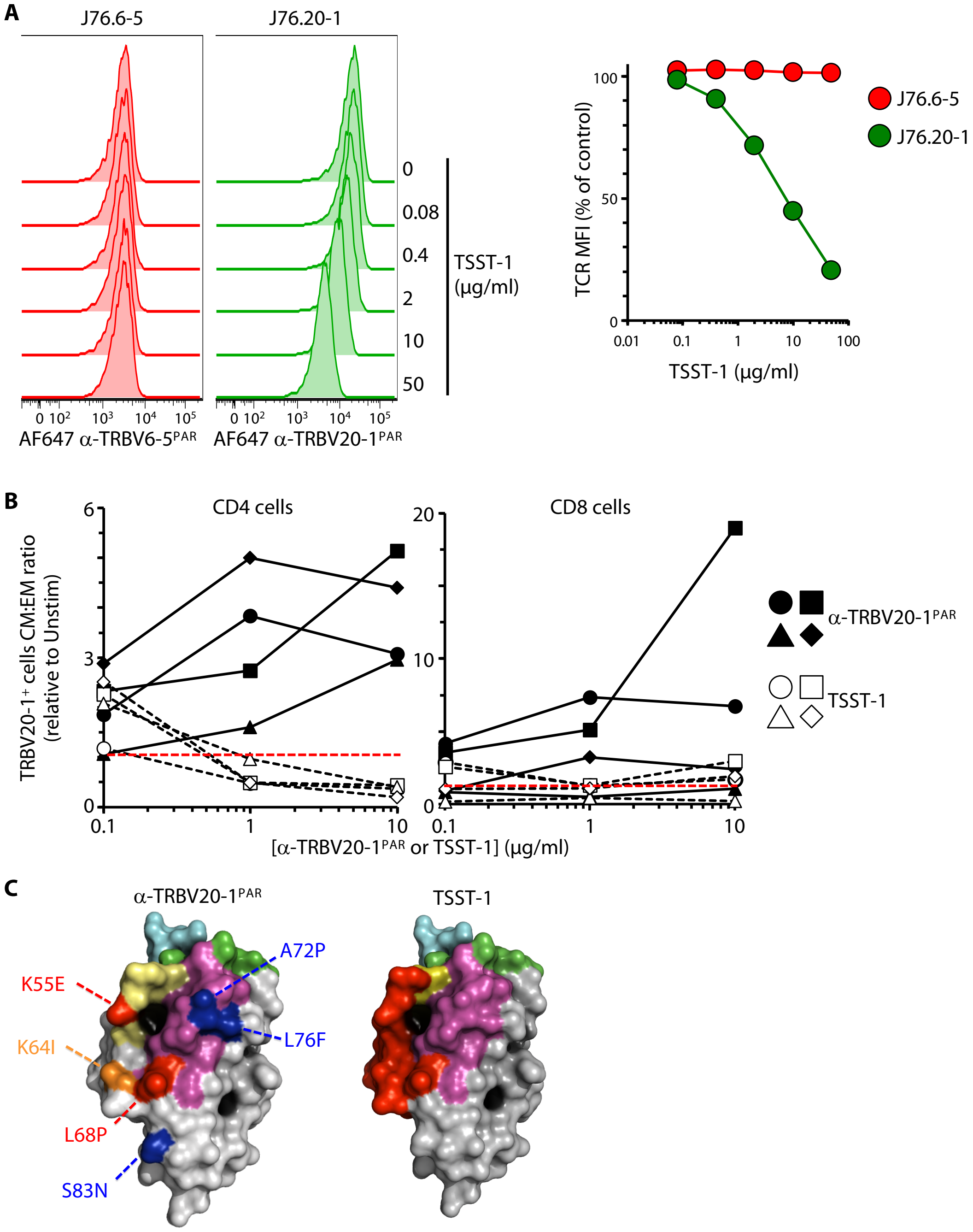

Supplementary Figure 8. Comparison of α-TRBV20-1 *versus* TSST-1.

A. J76 cells co-expressing a TRAV24^{+} TCRα chain and a TRBV6-5^{+} (J76.6-5) or TRBV20-1^{+} (J76.20-1) TCRβ chain were preincubated for 30 mins at 4˚C with the indicated doses of TSST-1, then stained with the corresponding α-TRBV antibody (flow histograms; α-TRBV6-5^{PAR}, left; α-TRBV20-1^{PAR}, right). Scatter plot shows the median fluorescence intensity (MFI) of TCR staining as the mean of triplicate stainings. Representative of two independent experiments. **B.** Summary data analysis of the CM:EM phenotype ratio after culture with α-TRBV antibodies (closed symbols) and superantigens (open symbols), relative to the Unstim baseline indicated by the red dashed line; data points above or below this line are indicative of a bias towards a CM or EM phenotype, respectively. Symbol shapes each correspond to one donor. Related to Fig 7A,B. C**.** Comparison of the epitopes mapped for α-TRBV20-1^{PAR} and binding site of TSST-1 onto TRBV20-1 (derived from (*72*) and (*73*)).

Supplementary Tables Legends:

Supplementary Table 1. Crystal structure data collection summary.

Data collection and refinement statistics for crystal structure determination of anti-TRBV6-5^{AM} antibody bound to TRBV6-5~TRAV12-3. Statistics for the highest resolution shell are shown in parentheses.

Supplementary Table 2. Quantification of cytokine production.

Complete cytokine secretion analysis data using the LegendPlex Human T_H and Anti-Virus Response panels, related to Fig 4C and Supplementary Fig 4B,C. Data are the mean of technical duplicates and expressed as ng per 10^5 T cells.

Supplementary Table 3. Differentially Expressed Genes analysis comparing Clusters 5 *versus* 0 and 7 *versus* 4.

Differentially Expressed Genes (DEG) analysis comparing $CD4^{+}$ T cells in Clusters 5 vs. 0 (enriched in α-TRBV6-5^{AM} vs. α-$CD3ε^{hSP34}$ stimulations, respectively) and $CD8^{+}$ T cells in Clusters 7 vs. 4 (enriched in α-TRBV6-5^{AM} vs. α-$CD3ε^{hSP34}$ stimulations, respectively). Adjusted p-values were determined by a Wilcoxon rank sum test with Bonferroni correction.

Supplementary Table 4. Differentially Expressed Genes analysis of α-TRBV-stimulated cells comparing targeted *versus* non-targeted cells.

Differentially Expressed Genes (DEG) analysis comparing CD4 or CD8 cells determined to express a TRBV segment targeted (TARG.; TRBV6-5, 6-1, 6-2, 10-3) vs. non-targeted (NON-TARG.; all other TRBV) by the α-TRBV6-5^{AM} antibody, derived only from the α-TRBV6-5^{AM} condition. Adjusted p-values were determined by a Wilcoxon rank sum test with Bonferroni correction.

Supplementary Table 5. Differentially Expressed Genes analysis of α-TRBV-stimulated cells comparing $CD25^{+}$ *versus* $CD25^{NEG}$ cells.

Differentially Expressed Genes (DEG) analysis comparing CD4 or CD8 cells determined to be $CD25^{+}$ vs. $CD25^{NEG}$, derived only from the α-TRBV6-5^{AM} condition. Adjusted p-values were determined by a Wilcoxon rank sum test with Bonferroni correction.

Supplementary Table 6. Gene expression analysis using NanoString.

Purified T cells from 3 donors (D1, D2, D3) were cultured for 7 days with the indicated plate-bound antibodies in complete media supplemented with IL-2 and IL-15. Cells were then stained for sorting of 4 subsets per sample, on the basis of CD4 or CD8 expression and staining by the α-TRBV6-5^{AM} antibody (6-5^{+}) or a combination of α-TRBV5-1, 12-3/4, 19, and 20-1 for the control (Ctrl, non-targeted by α-TRBV6-5^{AM}) subset. RNA was extracted immediately after sorting and gene expression (normalized counts) was determined by NanoString analysis using the Human CAR-T Characterization panel supplemented with (Panel Plus) probes for CD101, FCER1G, HOPX, ID3, ITGAE (CD103), KLF2, LGALS1 (Galectin-1), ZBED2, ZBTB7A (LRF), and ZNF683 (Hobit).

Supplementary Table 7. List of genes used to define the T_{RM} signature.

Genes used as the basis for determining the T_{RM} signature, from (*49*). Related to Supp Fig 6E.

Supplementary Table 8. List of antibodies and fluorescent reagents.

Name, supplier and catalogue number of the antibodies and fluorescent reagents used in this study.

REFERENCES

1. D. Mangani, D. Yang, A. C. Anderson, Learning from the nexus of autoimmunity and cancer. *Immunity* **56**, 256–271 (2023).

2. A. Sette, J. Sidney, S. Crotty, T Cell Responses to SARS-CoV-2. *Annu. Rev. Immunol.*. **41**, 343–373 (2023).

3. Y. D. Mahnke, T. M. Brodie, F. Sallusto, M. Roederer, E. Lugli, The who's who of T-cell differentiation: Human memory T-cell subsets. *Eur. J. Immunol.* **43**, 2797–2809 (2013).

4. M. Osman, S. L. Park, L. K. Mackay, Tissue-resident memory T (TRM) cells: Front-line workers of the immune system. *Eur. J. Immunol.*, **53**, e2250060 (2023).

5. F. Alfei, K. Kanev, M. Hofmann, M. Wu, H. E. Ghoneim, P. Roelli, D. T. Utzschneider, M. von Hoesslin, J. G. Cullen, Y. Fan, V. Eisenberg, D. Wohlleber, K. Steiger, D. Merkler, M. Delorenzi, P. A. Knolle, C. J. Cohen, R. Thimme, B. Youngblood, D. Zehn, TOX reinforces the phenotype and longevity of exhausted T cells in chronic viral infection. *Nature* **571**, 265–269 (2019).

6. M. Hashimoto, K. Araki, M. A. Cardenas, P. Li, R. R. Jadhav, H. T. Kissick, W. H. Hudson, D. J. McGuire, R. C. Obeng, A. Wieland, J. Lee, D. T. McManus, J. L. Ross, S. J. Im, J. Lee, J. X. Lin, B. Hu, E. E. West, C. D. Scharer, G. J. Freeman, A. H. Sharpe, S. S. Ramalingam, A. Pellerin, V. Teichgräber, W. J. Greenleaf, C. Klein, J. J. Goronzy, P. Umaña, W. J. Leonard, K. A. Smith, R. Ahmed, PD-1 combination therapy with IL-2 modifies CD8(+) T cell exhaustion program. *Nature* **610**, 173–181 (2022).

7. T. W. Wang, Y. Johmura, N. Suzuki, S. Omori, T. Migita, K. Yamaguchi, S. Hatakeyama, S. Yamazaki, E. Shimizu, S. Imoto, Y. Furukawa, A. Yoshimura, M. Nakanishi, Blocking PD-L1-PD-1 improves senescence surveillance and ageing phenotypes. *Nature* **611**, 358–364 (2022).

8. S. Becattini, D. Latorre, F. Mele, M. Foglierini, C. de Gregorio, A. Cassotta, B. Fernandez, S. Kelderman, T. N. Schumacher, D. Corti, A. Lanzavecchia, F. Sallusto, Functional heterogeneity of human memory CD4+T cell clones primed by pathogens or vaccines. *Science* **347**, 400–406 (2015).

9. L. Chen, D. B. Flies, Molecular mechanisms of T cell co-stimulation and co-inhibition. *Nat. Rev. Immunol.* **13**, 227–242 (2013).

10. M. A. Daniels, E. Teixeiro, J. Gill, B. Hausmann, D. Roubaty, K. Holmberg, G. Werlen, G. A. Holländer, N. R. J. Gascoigne, E. Palmer, Thymic selection threshold defined by compartmentalization of Ras/MAPK signalling. *Nature* **444**, 724–729 (2006).

11. T. Kawabe, J. Yi, J. Sprent, Homeostasis of naive and memory T lymphocytes. *Cold Spring Harb. Perspect. Biol.* **13**, (2021).

12. A. C. Hayday, P. Vantourout, The innate biologies of adaptive antigen receptors. *Annu. Rev. Immunol.* **38**, 487–510 (2020).

13. E. J. Sundberg, L. Deng, R. A. Mariuzza, TCR recognition of peptide/MHC class II complexes and superantigens. *Semin. Immunol.* **19**, 262–271 (2007).

14. M. Saline, K. E. J. Rödström, G. Fischer, V. Y. Orekhov, B. G. Karlsson, K. Lindkvist-Petersson, The structure of superantigen complexed with TCR and MHC reveals novel insights into superantigenic T cell activation. *Nat. Commun.* **1**, 119 (2010).

15. E. J. Sundberg, H. Li, A. S. Llera, J. K. McCormick, J. Tormo, P. M. Schlievert, K. Karjalainen, R. A. Mariuzza, Structures of two streptococcal superantigens bound to TCR beta chains reveal diversity in the architecture of T cell signaling complexes. *Structure* **10**, 687–699 (2002).

16. P. Dellabona, J. Peccoud, J. Kappler, P. Marrack, C. Benoist, D. Mathis, Superantigens interact with MHC class II molecules outside of the antigen groove. *Cell* **62**, 1115–1121 (1990).

17. J. D. Fraser, High-affinity binding of staphylococcal enterotoxins A and B to HLA-DR. *Nature* **339**, 221–223 (1989).

18. K. E. Rodstrom, K. Elbing, K. Lindkvist-Petersson, Structure of the superantigen staphylococcal enterotoxin B in complex with TCR and peptide-MHC demonstrates absence of TCR-peptide contacts. *J. Immunol.* **193**, 1998–2004 (2014).

19. D. Melandri, I. Zlatareva, R. A. G. Chaleil, R. J. Dart, A. Chancellor, O. Nussbaumer, O. Polyakova, N. A. Roberts, D. Wesch, D. Kabelitz, P. M. Irving, S. John, S. Mansour, P. A. Bates, P. Vantourout, A. C. Hayday, The γδTCR combines innate immunity with adaptive immunity by utilizing spatially distinct regions for agonist selection and antigen responsiveness. *Nat. Immunol.* **19**, 1352–1365 (2018).

20. C. R. Willcox, P. Vantourout, M. Salim, I. Zlatareva, D. Melandri, L. Zanardo, R. George, S. Kjaer, M. Jeeves, F. Mohammed, A. C. Hayday, B. E. Willcox, Butyrophilin-like 3 directly binds a human Vγ4+ T cell receptor using a modality distinct from clonally-restricted antigen. *Immunity* **51**, 813–825.e4 (2019).

21. L. M. Boyden, J. M. Lewis, S. D. Barbee, A. Bas, M. Girardi, A. C. Hayday, R. E. Tigelaar, R. P. Lifton, Skint1, the prototype of a newly identified immunoglobulin superfamily gene cluster, positively selects epidermal gammadelta T cells. *Nat. Genet.* **40**, 656–662 (2008).

22. R. Di Marco Barros, N. A. Roberts, R. J. Dart, P. Vantourout, A. Jandke, O. Nussbaumer, L. Deban, S. Cipolat, R. Hart, M. L. Iannitto, A. Laing, B. Spencer-Dene, P. East, D. Gibbons, P. M. Irving, P. Pereira, U. Steinhoff, A. Hayday, Epithelia use butyrophilin-like molecules to shape organ-specific γδ T cell compartments. *Cell* **167**, 203–218.e17 (2016).

23. A. Jandke, D. Melandri, L. Monin, D. S. Ushakov, A. G. Laing, P. Vantourout, P. East, T. Nitta, T. Narita, H. Takayanagi, R. Feederle, A. Hayday, Butyrophilin-like proteins display combinatorial diversity in selecting and maintaining signature intraepithelial γδ T cell compartments. *Nat. Commun.* **11**, 3769 (2020).

24. P. C. Johnson, J. F. Gainor, R. J. Sullivan, D. L. Longo, B. Chabner, Immune checkpoint inhibitors - the need for innovation. *N. Engl. J. Med.* **388**, 1529–1532 (2023).

25. D. K. Cole, F. Yuan, P. J. Rizkallah, J. J. Miles, E. Gostick, D. A. Price, G. F. Gao, B. K. Jakobsen, A. K. Sewell, Germ line-governed recognition of a cancer epitope by an immunodominant human T-cell receptor. *J. Biol. Chem.* **284**, 27281–27289 (2009).

26. M. Moreews, K. le Gouge, S. Khaldi-Plassart, R. Pescarmona, A. L. Mathieu, C. Malcus, S. Djebali, A. Bellomo, O. Dauwalder, M. Perret, M. Villard, E. Chopin, I. Rouvet, F. Vandenesch, C. Dupieux, R. Pouyau, S. Teyssedre, M. Guerder, T. Louazon, A. Moulin-Zinsch, M. Duperril, H. Patural, L. Giovannini-Chami, A. Portefaix, B. Kassai, F. Venet, G. Monneret, C. Lombard, H. Flodrops, J. M. de Guillebon, F. Bajolle, V. Launay, P. Bastard, S. Y. Zhang, V. Dubois, O. Thaunat, J. C. Richard, M. Mezidi, O. Allatif, K. Saker, M. Dreux, L. Abel, J. L. Casanova, J. Marvel, S. Trouillet-Assant, D. Klatzmann, T. Walzer, E. Mariotti-Ferrandiz, E. Javouhey, A. Belot, Polyclonal expansion of TCR Vbeta 21.3(+) CD4(+) and CD8(+) T cells is a hallmark of Multisystem Inflammatory Syndrome in Children. *Sci Immunol.* **6**, (2021).

27. L. Trautmann, M. Rimbert, K. Echasserieau, X. Saulquin, B̂... Neveu, J. Dechanet, V. Cerundolo, M. Bonneville, Selection of T cell clones expressing high-affinity public TCRs within Human cytomegalovirus-specific CD8 T cell responses. *J. Immunol.* **175**, 6123–6132 (2005).

28. Y. W. Choi, B. Kotzin, J. Lafferty, J. White, M. Pigeon, R. Kubo, J. Kappler, P. Marrack, A method for production of antibodies to human T-cell receptor beta-chain variable regions. *Proc. Natl. Acad. Sci. U.S.A.* **88**, 8357–8361 (1991).

29. S. Hori, T. Nomura, S. Sakaguchi, Control of regulatory T cell development by the transcription factor Foxp3. *Science* **299**, 1057–1061 (2003).

30. J. Sprent, C. D. Surh, Normal T cell homeostasis: The conversion of naive cells into memory-phenotype cells. *Nat. Immunol.* **12**, 478–484 (2011).

31. P. C. Beverley, Functional analysis of human T cell subsets defined by CD45 isoform expression. *Semin. Immunol.* **4**, 35–41 (1992).

32. F. Sallusto, D. Lenig, R. Forster, M. Lipp, A. Lanzavecchia, Two subsets of memory T lymphocytes with distinct homing potentials and effector functions. *Nature* **401**, 708–712 (1999).

33. J. J. Milner, H. Nguyen, K. Omilusik, M. Reina-Campos, M. Tsai, C. Toma, A. Delpoux, B. S. Boland, S. M. Hedrick, J. T. Chang, A. W. Goldrath, Delineation of a molecularly distinct terminally

differentiated memory CD8 T cell population. *Proc. Natl. Acad. Sci. U.S.A.* **117**, 25667–25678 (2020).

34. Y. Chen, J. Shen, M. Y. Kasmani, P. Topchyan, W. Cui, Single-cell transcriptomics reveals core regulatory programs that determine the heterogeneity of circulating and tissue-resident memory $CD8^+$ T cells. *Cells* **10**, 2143 (2021).

35. L. Xu, Y. Cao, Z. Xie, Q. Huang, Q. Bai, X. Yang, R. He, Y. Hao, H. Wang, T. Zhao, Z. Fan, A. Qin, J. Ye, X. Zhou, L. Ye, Y. Wu, The transcription factor TCF-1 initiates the differentiation of T(FH) cells during acute viral infection. *Nat. Immunol.* **16**, 991–999 (2015).

36. Y. S. Choi, J. A. Gullicksrud, S. Xing, Z. Zeng, Q. Shan, F. Li, P. E. Love, W. Peng, H. H. Xue, S. Crotty, LEF-1 and TCF-1 orchestrate T(FH) differentiation by regulating differentiation circuits upstream of the transcriptional repressor Bcl6. *Nat. Immunol.* **16**, 980–990 (2015).

37. S. Crotty, R. J. Johnston, S. P. Schoenberger, Effectors and memories: Bcl-6 and Blimp-1 in T and B lymphocyte differentiation. *Nat. Immunol.* **11**, 114–120 (2010).

38. D. Herndler-Brandstetter, H. Ishigame, R. Shinnakasu, V. Plajer, C. Stecher, J. Zhao, M. Lietzenmayer, L. Kroehling, A. Takumi, K. Kometani, T. Inoue, Y. Kluger, S. M. Kaech, T. Kurosaki, T. Okada, R. A. Flavell, $KLRG1^+$ Effector $CD8^+$ T Cells Lose KLRG1, Differentiate into All Memory T Cell Lineages, and Convey Enhanced Protective Immunity. *Immunity* **48**, 716–729.e8(2018).

39. J. J. Obar, E. R. Jellison, B. S. Sheridan, D. A. Blair, Q. M. Pham, J. M. Zickovich, L. Lefrançois, Pathogen-induced inflammatory environment controls effector and memory CD8+ T cell differentiation. *J. Immunol.* **187**, 4967–4978 (2011).

40. D. Wang, H. Diao, A. J. Getzler, W. Rogal, M. A. Frederick, J. Milner, B. Yu, S. Crotty, A. W. Goldrath, M. E. Pipkin, The transcription factor Runx3 establishes chromatin accessibility of cis-regulatory landscapes that drive memory cytotoxic T lymphocyte formation. *Immunity* **48**, 659–674.e6 (2018).

41. F. Cruz-Guilloty, M. E. Pipkin, I. M. Djuretic, D. Levanon, J. Lotem, M. G. Lichtenheld, Y. Groner, A. Rao, Runx3 and T-box proteins cooperate to establish the transcriptional program of effector CTLs. *J. Exp. Med.* **206**, 51–59 (2009).

42. T. L. Murphy, R. Tussiwand, K. M. Murphy, Specificity through cooperation: BATF-IRF interactions control immune-regulatory networks. *Nat. Rev. Immunol.* **13**, 499–509 (2013).

43. H. Seo, E. González-Avalos, W. Zhang, P. Ramchandani, C. Yang, C. W. J. Lio, A. Rao, P. G. Hogan, BATF and IRF4 cooperate to counter exhaustion in tumor-infiltrating CAR T cells. *Nat. Immunol.* **22**, 983–995 (2021).

44. K. Karwacz, E. R. Miraldi, M. Pokrovskii, A. Madi, N. Yosef, I. Wortman, X. Chen, A. Watters, N. Carriero, A. Awasthi, A. Regev, R. Bonneau, D. Littman, V. K. Kuchroo, Critical role of IRF1 and BATF in forming chromatin landscape during type 1 regulatory cell differentiation. *Nat. Immunol.* **18**, 412–421 (2017).

45. J. Mahnke, V. Schumacher, S. Ahrens, N. Käding, L. M. Feldhoff, M. Huber, J. Rupp, F. Raczkowski, H. W. Mittrücker, Interferon Regulatory Factor 4 controls T(H1) cell effector function and metabolism. *Sci. Rep.* **6**, 35521 (2016).

46. M. Vaeth, M. Maus, S. Klein-Hessling, E. Freinkman, J. Yang, M. Eckstein, S. Cameron, S. E. Turvey, E. Serfling, F. Berberich-Siebelt, R. Possemato, S. Feske, Store-operated Ca(2+) entry controls clonal expansion of T cells through metabolic reprogramming. *Immunity* **47**, 664–679.e6 (2017).

47. J. Cantor, M. Slepak, N. Ege, J. T. Chang, M. H. Ginsberg, Loss of T cell CD98 H chain specifically ablates T cell clonal expansion and protects from autoimmunity. *J. Immunol.* **187**, 851–860 (2011).

48. K. Man, S. S. Gabriel, Y. Liao, R. Gloury, S. Preston, D. C. Henstridge, M. Pellegrini, D. Zehn, F. Berberich-Siebelt, M. A. Febbraio, W. Shi, A. Kallies, Transcription factor IRF4 promotes CD8(+) T cell exhaustion and limits the development of memory-like T cells during chronic infection. *Immunity* **47**, 1129–1141.e5 (2017).

49. J. J. Milner, C. Toma, B. Yu, K. Zhang, K. Omilusik, A. T. Phan, D. Wang, A. J. Getzler, T. Nguyen, S. Crotty, W. Wang, M. E. Pipkin, A. W. Goldrath, Runx3 programs $CD8^+$ T cell residency in non-lymphoid tissues and tumours. *Nature* **552**, 253–257 (2017).

50. C. Li, B. Zhu, Y. M. Son, Z. Wang, L. Jiang, M. Xiang, Z. Ye, K. E. Beckermann, Y. Wu, J. W. Jenkins, P. J. Siska, B. G. Vincent, Y. S. Prakash, T. Peikert, B. T. Edelson, R. Taneja, M. H. Kaplan, J. C. Rathmell, H. Dong, T. Hitosugi, J. Sun, The transcription factor Bhlhe40 programs mitochondrial regulation of resident CD8(+) T cell fitness and functionality. *Immunity* **51**, 491–507.e7 (2019).

51. Y. Choi, J. A. Lafferty, J. R. Clements, J. K. Todd, E. W. Gelfand, J. Kappler, P. Marrack, B. L. Kotzin, Selective expansion of T cells expressing V beta 2 in toxic shock syndrome. *J. Exp. Med.* **172**, 981–984 (1990).

52. Y. W. Choi, B. Kotzin, L. Herron, J. Callahan, P. Marrack, J. Kappler, Interaction of staphylococcus aureus toxin "superantigens" with human T cells. *Proc. Natl. Acad. Sci. U.S.A.* **86**, 8941–8945 (1989).

53. E. Simpson, K. Takacs, D. M. Altmann, Thymic repertoire selection by superantigens: Presentation by human and mouse MHC molecules. *Thymus* **23**, 1–13 (1994).

54. X. Ren, W. Wen, X. Fan, W. Hou, B. Su, P. Cai, J. Li, Y. Liu, F. Tang, F. Zhang, Y. Yang, J. He, W. Ma, J. He, P. Wang, Q. Cao, F. Chen, Y. Chen, X. Cheng, G. Deng, X. Deng, W. Ding, Y. Feng, R. Gan, C. Guo, W. Guo, S. He, C. Jiang, J. Liang, Y. M. Li, J. Lin, Y. Ling, H. Liu, J. Liu, N. Liu, S. Q. Liu, M. Luo, Q. Ma, Q. Song, W. Sun, G. X. Wang, F. Wang, Y. Wang, X. Wen, Q. Wu, G. Xu, X. Xie, X. Xiong, X. Xing, H. Xu, C. Yin, D. Yu, K. Yu, J. Yuan, B. Zhang, P. Zhang, T. Zhang, J. Zhao, P. Zhao, J. Zhou, W. Zhou, S. Zhong, X. Zhong, S. Zhang, L. Zhu, P. Zhu, B. Zou, J. Zou, Z. Zuo, F. Bai, X. Huang, P. Zhou, Q. Jiang, Z. Huang, J. X. Bei, L. Wei, X. W. Bian, X. Liu, T. Cheng, X. Li, P. Zhao, F. S. Wang, H. Wang, B. Su, Z. Zhang, K. Qu, X. Wang, J. Chen, R. Jin, Z. Zhang, COVID-19 immune features revealed by a large-scale single-cell transcriptome atlas. *Cell* **184**, 1895–1913.e19 (2021).

55. J. B. Johnnidis, Y. Muroyama, S. F. Ngiow, Z. Chen, S. Manne, Z. Cai, S. Song, J. M. Platt, J. M. Schenkel, M. Abdel-Hakeem, J. C. Beltra, A. R. Greenplate, M. A. A. Ali, K. Nzingha, J. R. Giles, C. Harly, J. Attanasio, K. E. Pauken, B. Bengsch, M. A. Paley, V. T. Tomov, M. Kurachi, D. A. A. Vignali, A. H. Sharpe, S. L. Reiner, A. Bhandoola, F. B. Johnson, E. J. Wherry, Inhibitory signaling sustains a distinct early memory CD8(+) T cell precursor that is resistant to DNA damage. *Sci Immunol.* **6**, (2021).

56. R. Fonseca, T. N. Burn, L. C. Gandolfo, S. Devi, S. L. Park, A. Obers, M. Evrard, S. N. Christo, F. A. Buquicchio, C. A. Lareau, K. M. McDonald, S. K. Sandford, N. M. Zamudio, N. G. Zanluqui, A. Zaid, T. P. Speed, A. T. Satpathy, S. N. Mueller, F. R. Carbone, L. K. Mackay, Runx3 drives a CD8(+) T cell tissue residency program that is absent in CD4(+) T cells. *Nat. Immunol.* **23**, 1236–1245 (2022).

57. J. Strid, S. J. Roberts, R. B. Filler, J. M. Lewis, B. Y. Kwong, W. Schpero, D. H. Kaplan, A. C. Hayday, M. Girardi, Acute upregulation of an NKG2D ligand promotes rapid reorganization of a local immune compartment with pleiotropic effects on carcinogenesis. *Nat. Immunol.* **9**, 146–154 (2008).

58. M. Corcoran, M. Chernyshev, M. Mandolesi, S. Narang, M. Kaduk, K. Ye, C. Sundling, A. Färnert, T. Kreslavsky, C. Bernhardsson, M. Larena, M. Jakobsson, G. B. Karlsson Hedestam, Archaic humans have contributed to large-scale variation in modern human T cell receptor genes. *Immunity* **56**, 635–652.e6 (2023).

59. D. R. McKenzie, R. Hart, N. Bah, D. S. Ushakov, M. Muñoz-Ruiz, R. Feederle, A. C. Hayday, Normality sensing licenses local T cells for innate-like tissue surveillance. *Nat. Immunol.* **23**, 411–422 (2022).

60. G. Turchinovich, A. C. Hayday, Skint-1 identifies a common molecular mechanism for the development of Interferon-γ-Secreting versus interleukin-17-secreting γδ T cells. *Immunity* **35**, 59–68 (2011).

61. M. M. Karunakaran, C. R. Willcox, M. Salim, D. Paletta, A. S. Fichtner, A. Noll, L. Starick, A. Nöhren, C. R. Begley, K. A. Berwick, R. A. G. Chaleil, V. Pitard, J. Déchanet-Merville, P. A. Bates,

B. Kimmel, T. J. Knowles, V. Kunzmann, L. Walter, M. Jeeves, F. Mohammed, B. E. Willcox, T. Herrmann, Butyrophilin-2A1 directly binds germline-encoded regions of the Vγ9Vδ2 TCR and is essential for phosphoantigen sensing. *Immunity* **52**, 487–498.e6 (2020).

62. M. Rigau, S. Ostrouska, T. S. Fulford, D. N. Johnson, K. Woods, Z. Ruan, H. E. G. McWilliam, C. Hudson, C. Tutuka, A. K. Wheatley, S. J. Kent, J. A. Villadangos, B. Pal, C. Kurts, J. Simmonds, M. Pelzing, A. D. Nash, A. Hammet, A. M. Verhagen, G. Vairo, E. Maraskovsky, C. Panousis, N. A. Gherardin, J. Cebon, D. I. Godfrey, A. Behren, A. P. Uldrich, Butyrophilin 2A1 is essential for phosphoantigen reactivity by γδ T cells. *Science* **367**, eaay5516 (2020).

63. M. S. Davey, C. R. Willcox, S. Hunter, S. A. Kasatskaya, E. B. M. Remmerswaal, M. Salim, F. Mohammed, F. J. Bemelman, D. M. Chudakov, Y. H. Oo, B. E. Willcox, The human Vδ2+ T-cell compartment comprises distinct innate-like Vγ9+ and adaptive Vγ9- subsets. *Nat. Commun.* **9**, 1760 (2018).

64. P. Zareie, C. Szeto, C. Farenc, S. D. Gunasinghe, E. M. Kolawole, A. Nguyen, C. Blyth, X. Y. X. Sng, J. Li, C. M. Jones, A. J. Fulcher, J. R. Jacobs, Q. Wei, L. Wojciech, J. Petersen, N. R. J. Gascoigne, B. D. Evavold, K. Gaus, S. Gras, J. Rossjohn, N. L. la Gruta, Canonical T cell receptor docking on peptide-MHC is essential for T cell signaling. *Science* **372**, eabe9124 (2021).

65. R. J. Mallis, J. S. Duke-Cohan, D. K. das, A. Akitsu, A. M. Luoma, D. Banik, H. M. Stephens, P. W. Tetteh, C. D. Castro, S. Krahnke, R. E. Hussey, B. Lawney, K. N. Brazin, P. A. Reche, W. Hwang, E. J. Adams, M. J. Lang, E. L. Reinherz, Molecular design of the γδT cell receptor ectodomain encodes biologically fit ligand recognition in the absence of mechanosensing. *Proc. Natl. Acad. Sci. U.S.A.* **118**, e2023050118 (2021).

66. P. V. Afonine, N. W. Moriarty, M. Mustyakimov, O. V. Sobolev, T. C. Terwilliger, D. Turk, A. Urzhumtsev, P. D. Adams, FEM: Feature-enhanced map. *Acta Crystallogr. D Biol. Crystallogr.* **71**, 646–666 (2015).

67. M. Munoz-Ruiz, Irma Pujol-Autonell, H. Rhys, H. M. Long, M. Greco, M. Peakman, T. Tree, A. C. Hayday, F. D. Rosa, Tracking immunodynamics by identification of S-G(2)/M-phase T cells in human peripheral blood. *J. Autoimmun.* **112**, 102466 (2020).

68. J. Le Nours, T. Praveena, D. G. Pellicci, N. A. Gherardin, F. J. Ross, R. T. Lim, G. S. Besra, S. Keshipeddy, S. K. Richardson, A. R. Howell, S. Gras, D. I. Godfrey, J. Rossjohn, A. P. Uldrich, Atypical natural killer T-cell receptor recognition of CD1d-lipid antigens. *Nat. Commun.* **7**, 10570 (2016).

69. D. Dong, L. Zheng, J. Lin, B. Zhang, Y. Zhu, N. Li, S. Xie, Y. Wang, N. Gao, Z. Huang, Structural basis of assembly of the human T cell receptor-CD3 complex. *Nature* **573**, 546–552 (2019).

70. M. C. Raman, P. J. Rizkallah, R. Simmons, Z. Donnellan, J. Dukes, G. Bossi, G. S. Le Provost, P. Todorov, E. Baston, E. Hickman, T. Mahon, N. Hassan, A. Vuidepot, M. Sami, D. K. Cole, B. K. Jakobsen, Direct molecular mimicry enables off-target cardiovascular toxicity by an enhanced affinity TCR designed for cancer immunotherapy. *Sci. Rep.* **6**, 18851 (2016).

71. J. R. Devlin, J. A. Alonso, C. M. Ayres, G. L. J. Keller, S. Bobisse, C. W. Vander Kooi, G. Coukos, D. Gfeller, A. Harari, B. M. Baker, Structural dissimilarity from self drives neoepitope escape from immune tolerance. *Nat. Chem. Biol.* **16**, 1269–1276 (2020).

72. S. B. Eckle, R. W. Birkinshaw, L. Kostenko, A. J. Corbett, Hamish E G Mc William, R. Reantragoon, Z. Chen, N. A. Gherardin, T. Beddoe, L. Liu, O. Patel, B. Meehan, D. P. Fairlie, J. A. Villadangos, D. I. Godfrey, L. Kjer-Nielsen, J. M. Cluskey, J. Rossjohn, A molecular basis underpinning the T cell receptor heterogeneity of mucosal-associated invariant T cells. *J. Exp. Med.* **211**, 1585–1600 (2014).

73. B. Moza, A. K. Varma, R. A. Buonpane, P. Zhu, C. A. Herfst, M. J. Nicholson, A. K. Wilbuer, N. P. Seth, K. W. Wucherpfennig, J. K. McCormick, D. M. Kranz, E. J. Sundberg, Structural basis of T-cell specificity and activation by the bacterial superantigen TSST-1. *EMBO J.* **26**, 1187–1197 (2007).
